# (+)-Lipoic acid reduces mitochondrial unfolded protein response and attenuates oxidative stress and aging in an in vitro model of non-alcoholic fatty liver disease

**DOI:** 10.1186/s12967-024-04880-x

**Published:** 2024-01-20

**Authors:** Lucia Longhitano, Alfio Distefano, Nicolò Musso, Paolo Bonacci, Laura Orlando, Sebastiano Giallongo, Daniele Tibullo, Simona Denaro, Giuseppe Lazzarino, Jessica Ferrigno, Anna Nicolosi, Amer M. Alanazi, Federico Salomone, Emanuela Tropea, Ignazio Alberto Barbagallo, Vincenzo Bramanti, Giovanni Li Volti, Giacomo Lazzarino, Daniele Torella, Angela Maria Amorini

**Affiliations:** 1 Department of Biomedical and Biotechnological Sciences, University of Catania, 95123 Catania, Italy; 2 Hospital Pharmacy Unit, Ospedale Cannizzaro, 95125 Catania, Italy; 3 Pharmaceutical Biotechnology Laboratory, Department of Pharmaceutical Chemistry, College of Pharmacy, King Saud University, 11451 Riyadh, Saudi Arabia; 4 Division of Gastroenterology, Ospedale Di Acireale, Azienda Sanitaria Provinciale Di Catania, Catania, Italy; 5 U.O.S. Laboratory Analysis, Maggiore “Nino Baglieri” Hospital - ASP Ragusa, 97015 Modica (RG), Italy; 6 UniCamillus-Saint Camillus International University of Health Sciences, Via Di Sant’Alessandro 8, 00131 Rome, Italy; 7 Department of Experimental and Clinical Medicine, Magna Graecia University, Catanzaro, Italy

**Keywords:** Non-alcoholic fatty liver disease, Mitochondrial dysfunction, Unfolded protein re-sponse, Oxidative stress

## Abstract

**Background:**

Non-alcoholic fatty liver disease (NAFLD) is a liver disorder characterized by the ac-cumulation of fat in hepatocytes without alcohol consumption. Mitochondrial dysfunction and endoplasmic reticulum (ER) stress play significant roles in NAFLD pathogenesis. The unfolded protein response in mitochondria (UPRmt) is an adaptive mechanism that aims to restore mitochondrial protein homeostasis and mitigate cellular stress. This study aimed to investigate the effects of ( +)-Lipoic acid (ALA) on UPRmt, inflammation, and oxidative stress in an in vitro model of NAFLD using HepG2 cells treated with palmitic acid and oleic acid to induce steatosis.

**Results:**

Treatment with palmitic and oleic acids increased UPRmt-related proteins HSP90 and HSP60 (heat shock protein), and decreased CLPP (caseinolytic protease P), indicating ER stress activation. ALA treatment at 1 μM and 5 μM restored UPRmt-related protein levels. PA:OA (palmitic acid:oleic acid)-induced ER stress markers IRE1α (Inositol requiring enzyme-1), CHOP (C/EBP Homologous Protein), BIP (Binding Immunoglobulin Protein), and BAX (Bcl-2-associated X protein) were significantly reduced by ALA treatment. ALA also enhanced ER-mediated protein glycosylation and reduced oxidative stress, as evidenced by decreased GPX1 (Glutathione peroxidase 1), GSTP1 (glutathione S-transferase pi 1), and GSR (glutathione-disulfide reductase) expression and increased GSH (Glutathione) levels, and improved cellular senescence as shown by the markers β-galactosidase, γH2Ax and Klotho-beta.

**Conclusions:**

In conclusion, ALA ameliorated ER stress, oxidative stress, and inflammation in HepG2 cells treated with palmitic and oleic acids, potentially offering therapeutic benefits for NAFLD providing a possible biochemical mechanism underlying ALA beneficial effects.

**Graphical Abstract:**

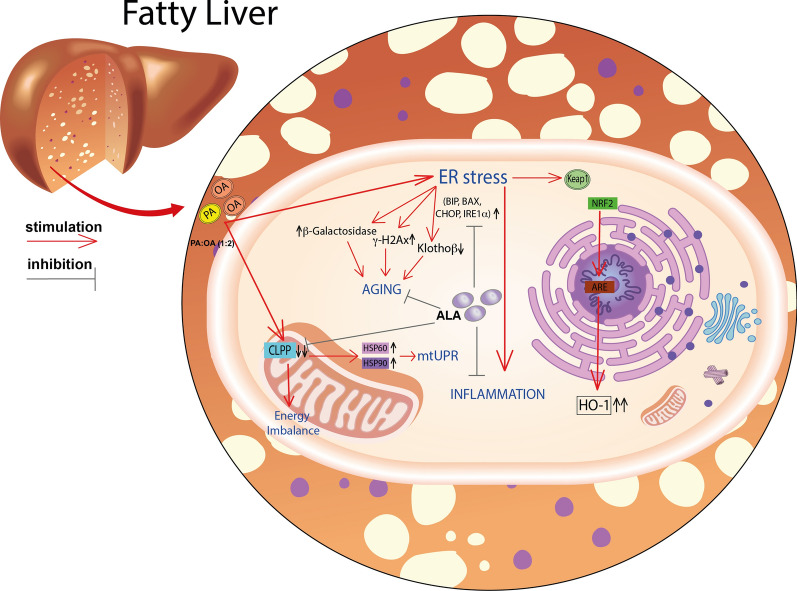

**Supplementary Information:**

The online version contains supplementary material available at 10.1186/s12967-024-04880-x.

## Introduction

Non-alcoholic fatty liver disease (NAFLD) is a spectrum of liver disorders. It is defined by the presence of steatosis in more than 5% of hepatocytes with little or no alcohol consumption [1].NAFLD is closely associated with obesity, insulin resistance, and other metabolic abnormalities [2, 3] and is characterized by an imbalance between lipid uptake, synthesis, oxidation, and export, leading to fat accumulation in the liver and subsequent mitochondrial dysfunction and stress [4]. Mitochondrial dysfunction in turn, triggers the unfolded protein response in mitochondria (UPRmt), which is a cellular stress response pathway activated following impaired protein folding within the mitochondria and directed at restoring mitochondrial protein homeostasis by promoting the expression of specific genes involved in mitochondrial protein folding, degradation, and quality control [5]. In addition, this pathway also regulates mitochondrial biogenesis and the removal of damaged mitochondria through mitophagy. Previous studies have demonstrated an association between NAFLD and UPRmt activation, as evidenced by the upregulation of UPRmt-related genes and proteins in NAFLD patients and animal models [6–8]. This suggests that UPRmt activation may represent an adaptive mechanism to restore mitochondrial protein homeostasis and mitigate cellular stress in NAFLD [7, 9–11]. However, it is important to note that prolonged or severe endoplasmic reticulum (ER) stress, such it occurs in NAFLD, can contribute to UPR dysfunction and liver injury [12, 13]. Consistently, elevated ER stress markers have been observed in the livers of individuals with NAFLD, and it can disrupt lipid metabolism, promote inflammation, and contribute to the progression of NAFLD to more severe forms [14]. Targeting ER stress has emerged as a potential therapeutic approach for NAFLD. Strategies to alleviate ER stress and improve ER function include the use of chemical chaperones and pharmacological agents that modulate the UPR signaling pathways [15]. Alpha-lipoic acid, in particular, has been reported to influence the expression of UPRmt-related genes and showed promise in experimental models by upregulating heat shock proteins and molecular chaperones involved in mitochondrial protein folding and quality control [16, 17]. Consistently, a recent controlled randomized trial showed that Alpha-lipoic acid supplementation in obese patients with NAFLD improved metabolic parameters and liver steatosis [18, 19]. This suggests that alpha-lipoic acid may contribute to maintaining mitochondrial homeostasis and mitigating mitochondrial stress in NAFLD. However, the exact role of UPRmt in NAFLD pathogenesis is not fully understood, and it can have both beneficial and detrimental effects depending on the context and severity of mitochondrial dysfunction. Therefore, the aim of the present study was to evaluate the role of UPRmt in an in vitro model of NAFLD and investigate the potential beneficial effects of lipoic acid on this pathophysiological pathway.

## Materials and methods

### Cell culture and pharmacological treatments

HepG2 cells (American Type Culture Collection, Manassas, VA, USA) were a kind gift from Prof. Maurizio Parola of the University of Turin. Briefly, low-passage cells were grown in DMEM (Sigma-Aldrich, Milan, Italy) supplemented with 10% FBS (EuroClone, Milan, Italy), 100 U/mL penicillin (Life Technologies, Milan, Italy), and 100 µg/mL streptomycin (Life Technologies) at 37 °C in a humidified incubator in an atmosphere of 95% air and 5% CO2. Upon reaching 80–90% confluency, to induce steatosis, HepG2 cells were pre-treated for 24 h as follows: HepG2 + vehicle (bovine serum albumin (BSA) 5%); HepG2 + PA:OA (BSA 5% + palmitic acid 250 µM and oleic acid 500 µM); after 24 h cells were treated for 6 or 24 h as follows: HepG2 + PA:OA + ɑ-lipoic acid (BSA 5% + palmitic acid 250 µM and oleic acid 500 µM + ɑ-lipoic acid 1 and 5 µM). (Fig. S1) Cell culture was tested for possible mycoplasma contamination before all the experimental procedures with a (PlasmoTest™, rep-pt1, InvivoGen) according to manufacturer’s protocol.

### Western Blot analysis

Briefly, for western blot analysis, 50 μg of proteins were loaded onto a 12% poly-acrylamide gel Mini- PROTEAN^®^ TGXTM (BIO-RAD, Milan, Italy). Electro-transfer to nitrocellulose membrane was obtained through Trans- Blot^®^ TurboTM (BIO-RAD), using Trans-Blot^®^ SE Semi-Dry Transfer Cell (BIO-RAD) [20, 21]. Membranes were blocked in Odyssey Blocking Buffer (Licor, Milan, Italy), according to the manufacturer’s protocol. After blocking, membranes were washed three times in PBS for 5 min and incubated with primary antibodies against HSP90 (heat shock protein 90) (1:500, ab203085, ABCAM), HSP60 (heat shock protein 60) (1:500, ab190828, ABCAM), CLPP (Caseinolytic Mitochondrial Matrix Peptidase Proteolytic Subunit) (1:500, Cat #PA5-52722, Invitrogen) and HO-1 (heme oxygenase 1) (1:1000, ab52947, ABCAM), overnight at 4 ℃. The next day, membranes were washed three times in PBS (Phosphate buffered saline) for 5 min and incubated with anti-rabbit IRDye700CW secondary antibodies (1:5000, Licor) in PBS/0.5% Tween-20 for 1 h at room temperature. All the antibodies were diluted in Odyssey Blocking Buffer. The obtained blots were visualized by Odyssey Infrared Imaging Scanner (Licor, Milan, Italy). Densitometric analysis was used for protein levels quantification, normalizing data to protein levels of β-actin (1:2000, ab8229, ABCAM).

### Real‐time PCR and RNAseq for gene expression analysis

Two identical plates of HepG2 cells were pre-treated with PA:OA to induce steatosis and, in the following 6 h, treated with ɑ-lipoic acid. RNA for Real-Time PCR was extracted from one of the plates using Trizol® reagent (Invitrogen, Carlsbad, CA, USA). First-strand complementary DNA (cDNA) was then synthesized with a reverse transcription reagent from Applied Biosystems (Foster City, CA, USA). Quantitative real-time PCR (qRT-PCR) was performed in StepOne Fast Real-Time PCR System (Applied Bio-systems) using the SYBR Green PCR MasterMix (Life Technologies, Monza, Italy). The specific PCR products were detected with SYBR Green fluorescence. The relative messenger RNA (mRNA) expression level was calculated by the threshold cycle (Ct) value of each PCR product and normalized with that of actin using a comparative 2 − DDCt method. The sequences of the primers used are presented in Table 1.

**Table 1:** Primer sequences and genes of interest

Gene	Forward 3′ – > 5′	Reverse 3′ – > 5′	Accession Number
*HMOX1*	TGTTGGAGCCACTCTGTTCC	GCTCAAAAACCACCCCAACC	NM_002133.3
*IRE1alpha*	CTCAGAGACAGCGCGAGTAG	ATCTCAGCCTAGCTGTCCCA	NM_001433.5
*CHOP*	ATGAACGGCTCAAGCAGGAA	GGGAAAGGTGGGTAGTGTGG	NM_001195053.1
*BIP*	CACTCCTGAAGGGGAACGTC	TCAAAGACCGTGTTCTCGGG	NM_005347.5
*BAX*	ATGGACGGGTCCGGGG	GGAAAAAGACCTCTCGGGGG	NM_001291428.2
*GSR*	AGGCTTCCTGCTGCTTCTG	CAACATTCACGCAAGTGCCA	NM_000637.5
*GSTP1*	AAGTTCCAGGACGGAGACCT	GCTGGTCCTTCCCATAGAGC	NM_000852.4
*GLCL*	ACTTCATTTCCCAGTACCTTAACA	CCGGCTTAGAAGCCCTTGAA	NM_001197115.2
*IL8*	TCTGCAGCTCTGTGTGAAGG	TTCTCAGCCCTCTTCAAAAACT	NM_000584.4
*β-Actin*	CCTTTGCCGATCCGCCG	AACATGATCTGGGTCATCTTCTCGC	NM_001101.5

The RNA for RNA sequencing has been extracted from the second plate following the manufacturer’s instructions of the Qiamp RNeasy Mini Kit (Cat. 74104, Qiagen, Hilden, Germany); the integrity and the quantification of the RNA were attested using Agilent RNA 6000 Nano Kit (Cat. 5067–1511, Agilent, Santa Clara, CA 95051, USA) on a 2100 Bioanalyzer Instrument (Cat. G2939BA, Agilent, Santa Clara, CA 95051, USA) and also using QubitTM RNA HS Assay Kit (Cat. 2390601, Invitrogen, Eugene, Oregon, USA) on a Qubit 4 Fluorometer instrument (Cat. Q33238, Invitrogen, Eugene, Oregon, USA). The samples and their quantifications are given in Additional file 6: Table S1.

#### Libraries preparation and sequencing

The libraries have been prepared following the manufacturer's instructions provided by the protocol generated by the website https://support.illumina.com/custom-protocol-selector.html and specifying the following supported combinations (Table 2)

**Table 2:** Supported combinations provided for the generation of the Illumina pro-tocol followed by the preparation of the libraries

Sequencing instrument	MiSeq
Library Preparation Kit	AmpliSeq for Illumina Custom and Community Panels
Input Material	Only RNA protocol
Indexing	Dual Indexing
Reagent Kits	MiSeq Reagent Kit v3

The RNA input used was 100 ng for all samples. The preparation was carried out by the AmpliSeq TM cDNA Synthesis for Illumina kit (Cat. 20022654, Illumina Inc., San Diego, California, USA) for retrotranscription, the AmpliSeq TM Library PLUS for Illumina (Cat. 20019102, Illumina^®^ Inc., San Diego, California, USA) for preparation and the AmpliSeq TM CD Indexes, Set A for Illumina^®^ (96 Indexes, 96 Samples) (Cat. 20019105, Illumina^®^ Inc., San Diego, California, USA) for sample indexing. The custom panel was designed using the Illumina DesignStudio Assay Design Tool (Illumina^®^ Inc., San Diego, California, USA), for each sample were sequenced 7186 bp. This provided the sequencing of 56 genes parts (Table 3). The denaturing and dilution of libraries were performed following the “Denature and Dilute Libraries Guide” protocol provided by Illumina^®^ (Document # 15039740 v10). Finally, sequencing was performed using the MiSeq Reagent Kits v3 (Cat. 15043895, Illumina^®^ Inc., San Diego, California, USA) on a MiSeq Instrument (Cat. SY-410–1003, Illumina^®^ Inc., San Diego, California, USA). Bioinformatic analysis, Different Expression Gene (DEG) and Statistical Analysis were carried out using QIAGEN CLC Genomics Workbench (Qiagen, Hilden, Germany).

**Table 3:** Genes included in the custom panel designed for RNA sequencing

*ABCC1* (ATP Binding Cassette Subfamily C Member 1)	*CX3CR1* (C-X3-C Motif Chemokine Receptor 1)	*IL4* (Interleukin 4)	*NQO1* (NAD(P)H Quinone Dehydrogenase 1)
*ABCC2* (ATP Binding Cassette Subfamily C Member 2)	*CYBB* (Cytochrome B-245 Beta Chain)	*IL6* (Interleukin 6)	*PARK7* (Parkinsonism Associated Deglycase)
*BAX* (BCL2 Associated X, Apoptosis Regulator)	*GCLC* (Glutamate-Cysteine Ligase Catalytic Subunit)	*IL8* (Interleukin 8)	*PRDX1* (Peroxiredoxin 1)
*BCL2* (BCL2 Apoptosis Regulator)	*GCLM* (Glutamate-Cysteine Ligase Modifier Subunit)	*JUN* (Jun Proto-Oncogene, AP-1 Transcription Factor Subunit)	*PRDX3* (Peroxiredoxin 3)
*CAT* (catalase)	*GPX1*(Glutathione Peroxidase 1)	*KCNK13* (Potassium Two Pore Domain Channel Subfamily K Member 13)	*SOD1* (Superoxide Dismutase 1)
*CCL2* (C–C Motif Chemokine Ligand 2)	*GSTP1* (Glutathione S-Transferase Pi 1)	*KEAP1* (Kelch Like ECH Associated Protein 1)	*SOD2* (Superoxide Dismutase 2)
*CCL5* (C–C Motif Chemokine Ligand 5)	*HMBS* (Hydroxymethylbilane Synthase)	*LRP1* (LDL Receptor Related Protein 1)	*SRXN1* (Sulfiredoxin 1)
*CHRNA2* (Cholinergic Receptor Nicotinic Alpha 2 Subunit)	*HMOX1* (Heme Oxygenase 1)	*MMP2* (Matrix Metallopeptidase 2)	*TGFB1* (Transforming Growth Factor Beta 1)
*CHRNA4* (Cholinergic Receptor Nicotinic Alpha 4 Subunit)	*IDE* (Insulin Degrading Enzyme)	*MMP9* (Matrix Metallopeptidase 9)	*TGFBR2* (Transforming Growth Factor Beta Receptor 2)
*CHRNA7* (Cholinergic Receptor Nicotinic Alpha 7 Subunit)	*IFNG* (Interferon Gamma)	*MRPL13* (Mitochondrial Ribosomal Protein L13)	*TNF* (Tumor Necrosis Factor)
*CHRNB2* (Cholinergic Receptor Nicotinic Beta 2 Subunit)	*IL10* (Interleukin 10)	*NFE2L2* (NFE2 Like BZIP Transcription Factor 2)	*TXN* (Thioredoxin)
*COX2* (Cyclooxygenase-2)	*IL17A* (Interleukin 17A)	*NFKB1* (Nuclear Factor Kappa B Subunit 1)	*TXN2* (Thioredoxin 2)
*CSF2* (Colony Stimulating Factor 2)	*IL1B* (Interleukin 1 beta)	*NOS2* (Nitric Oxide Synthase 2)	*TXNRD1* (Thioredoxin Reductase 1)
*CSF3* (Colony Stimulating Factor 3)	*IL1RN* (Interleukin 1 Receptor Antagonis)	*NOX1* (NADPH Oxidase 1)	*VEGFB* (Vascular Endothelial Growth Factor B)

### Immunocytochemical analysis

After washing with PBS, cells were fixed in 4% paraformaldehyde (category no. 1004968350 Sigma-Aldrich, Milan, Italy) for 20 min at room temperature. Subsequently, cells were incubated with antibodies against Nitrotyrosine (MAB3248), Nrf2 (Nuclear factor erythroid 2-related factor 2) (ab62352), γH2AX (ab22551) and klotho-beta (AF5889) at dilution 1:200, overnight at 4 °C. The next day, cells were washed three times in PBS for 5 min and were incubated with secondary antibody for 1 h. Cells were washed three times in PBS for 5 min and nuclei were stained by NucBlue (two drops per mL) (Thermo Fisher Scientific, Milan, Italy) for 15 min at 37 °C, according to the manufacturer’s instructions. The fluorescent images were obtained using Operetta (PerkinElmer, MA, USA). In particular, for Nrf2 translocation measurement and γH2AX quantification, we used Harmony software (PerkinElmer, MA, USA) following nuclear segmentation using the DAPI channel.

### HPLC analysis of GSH and UDP-derivatives

At the end of incubation under the different experimental conditions (controls, PA:O, PA:O + ALA), cells (3 × 106, n = 6 replicates) were washed twice with large volumes of ice-cold 10 mM PBS at pH 7.4 and then centrifuged at 1860 × g for 5 min at 4 °C. Cell pellets were vigorously mixed with 1 ml of ice-cold, nitrogen-saturated, deproteinizing solution (10 mM KH_2_PO_4_ + HPLC-grade CH_3_CN, pH 7.4, 1:3 v:v) and centrifuged at 20,690 × g, for 10 min at 4 °C. The supernatants were mixed with two volumes of HPLC-grade chloro-form, centrifuged (20,690 g, for 10 min at 4 °C) and the upper aqueous phase was re-covered and used for the analysis of GSH, UDP-galactose (UDP-Gal), UDP-glucose (UDP-Glc), UDP-N-acetyl-galactose (UDP-GalNac) and UDP-N-acetyl-glucose (UDP-GlcNac), according to an ion-pairing HPLC method described in detail elsewhere [22, 23].

### Cytofluorimetric analysis of mitoROS and mitochondrial depolarization

A membrane potential probe, the DiOC2(3) (3,3’-Diethyloxacarbocyanine Iodide), was used to evaluate the mitochondrial membrane potential. Cells were incubated with 10 μM DiOC2(3) (Thermo Fisher Scientific, Milan, Italy) for 30 min at 37 °C, washed twice and resuspended in PBS for flow cytometry analysis. In order to measure changes in the mitochondrial ROS (mitoROS), cells were reacted with mitoSOX probe (Thermo Fisher Scientific, Milan, Italy), according to the manufacturer’s instructions. After being washed twice, labelled mitochondria were analyzed by flow cytometry.The intensity of fluorescence of DiOC2(3) was detected using the MACSQuant Analyzer, as previously de-scribed [23, 24].

### Statistical Analysis

Statistical analysis was performed using GraphPad Prism Software, version 9.0 (GraphPad Software Inc., California, USA, RRID: rid_000081). For comparison of n ≥ 3 groups, one-way analysis of variance (ANOVA) for multiple comparisons, followed by either the Tukey-test or corrected for the false discovery rate according to the two-stage linear step-up procedure of Benjamini, Krieger and Yekutieli, was used. Data are ex-pressed as mean ± SD, unless otherwise stated and, p-values < 0.05 were considered statistically significant.

## Results

### ALA restores the effect of palmitic acid/oleic acid on HepG2 ER stress and unfolded protein response.

**Fig. 1:**
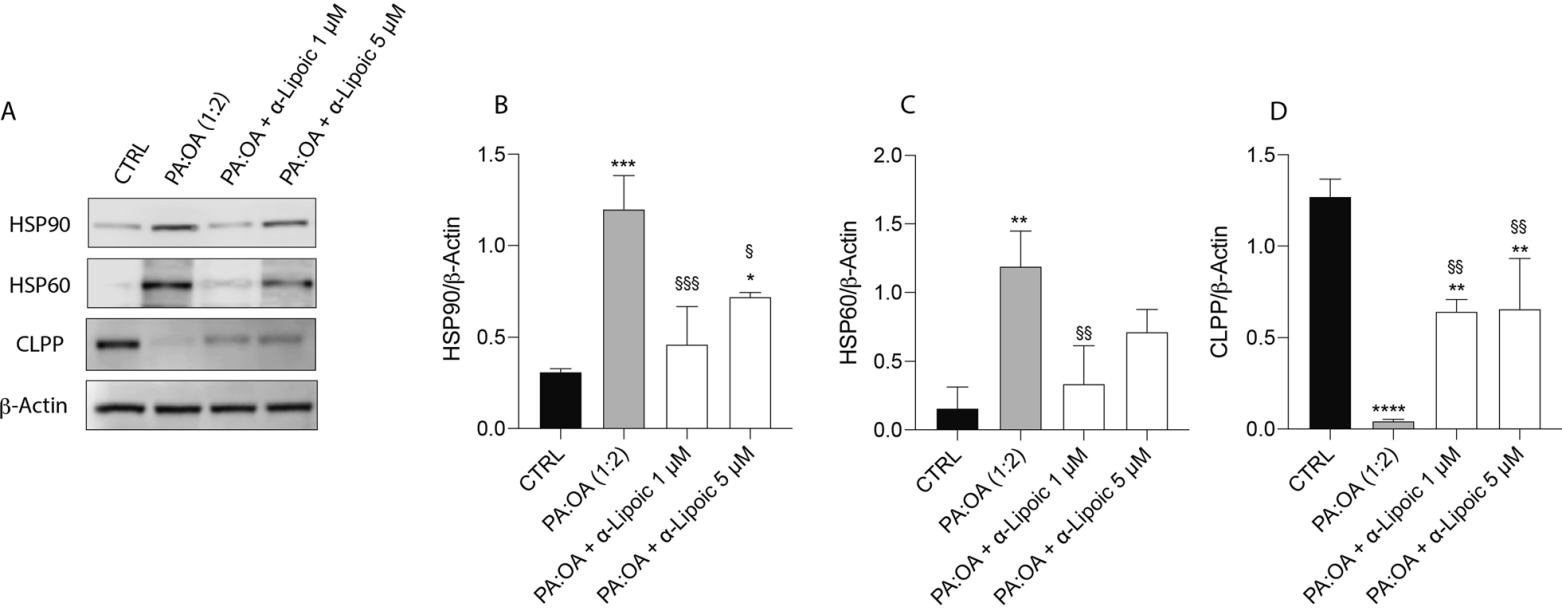
ALA reverses the effect of palmitic acid/oleic acid on unfolded protein response in HepG2 cells. **A** Western blot analysis of HSP90, HSP60 and CLPP proteins. **B** Densitometry analysis of HSP90. **C** Densitometry analysis of HSP60. **D** Densitometry analysis of CLPP. β‐Actin protein was used as total protein loading reference. Values represent the mean ± SD of experiments performed in quadruplicate. * vs CTRL (*p < 0.05, **p < 0.01, ***p < 0.001, ****p < 0.0001); ^§^ vs PA:OA (^§^ p < 0.05, ^§§^ p < 0.01, ^§§§^ p < 0.001)

We, first, analyzed the effect of PA:OA (palmitic acid/oleic acid) in HepG2 unfolded protein response (UPR). Our data showed that PA:OA induced a significant increase in HSP90 (Fig. 1A, B) and HSP60 (Fig. 1A, C) (p < 0.01) and a significant decrease in CLPP (Fig. 1 A, D) (p < 0.0001) protein expression, compared to control HepG2 cells. Thus, we evaluated the effect of ALA supplementation in PA:OA-treated HepG2 cells. Interestingly, our results showed that ALA treatment, both at 1 and 5 µM concentration, was able to restore the effect of PA:OA, resulting in a significant decrease in HSP90 and HSP60 protein expression, but the treatment with ALA alone at both concentrations results in a significant increase in HSP90, HSP60 and CLPP protein expression (Additional file 1: Fig. S1). In order to further confirm the involvement of the mitochondrial unfolded protein response, we also evaluated the expression of CLPP, which is a mammalian quality control protease playing a major role in such process. Interestingly, our results showed a significant decrease in CLPP protein expression following PA:OA treatment, which was reversed following ALA treatment at both concentrations (Fig. 1A–D).

**Fig. 2:**
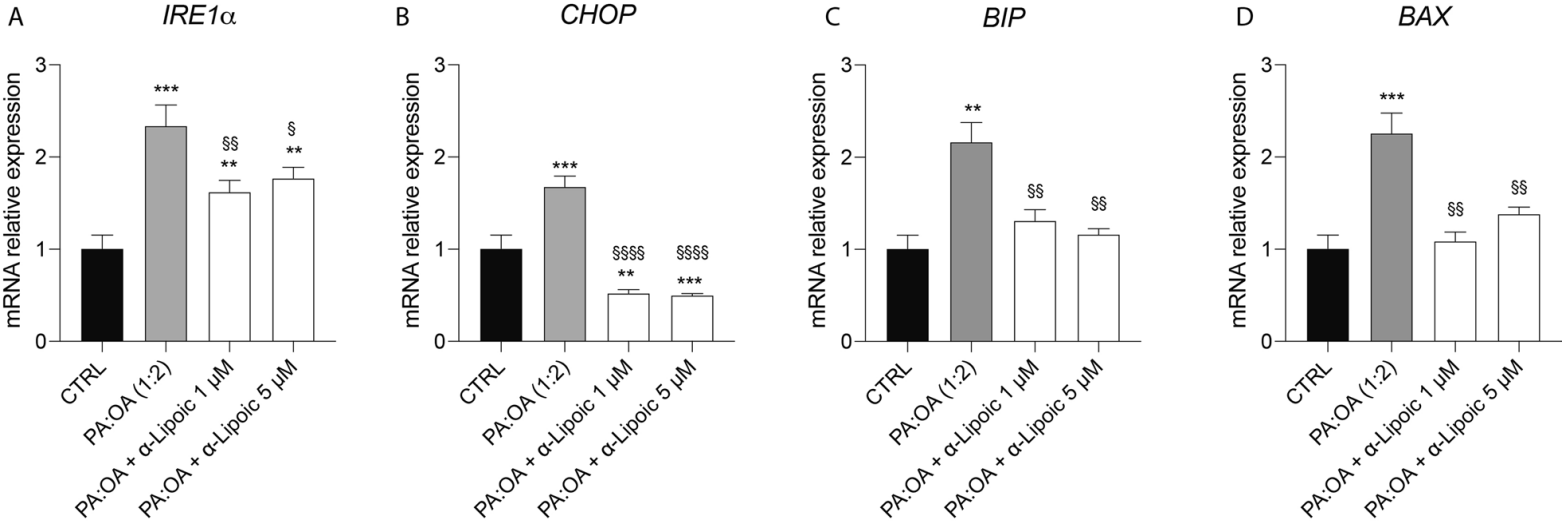
ALA reverses the effect of palmitic acid/oleic acid on ER stress in HepG2 cells. mRNA expression levels of **A** IRE1α, **B** CHOP, **C** BIP and **D** BAX. β-Actin gene was used as housekeeping gene. Values represent the mean ± SD of experiments performed in quadruplicate. * vs CTRL (** p < 0.01, *** p < 0.001); ^§^ vs PA:OA (^§^ p < 0.05, ^§§^ p < 0.01, ^§§§§^ p < 0.0001)

Given the effect of ALA on UPR of PA:OA-treated HepG2 cells, we studied the effect on endoplasmic reticulum (ER) stress. To this aim, we analyzed the expression of the main ER stress markers, as well as of the most relevant metabolites (UDP-Gal. UDP-Glc, UDP-GalNac, UDP-GlcNac) of the hexosamine biosynthetic pathway (HBP) of paramount importance in the crucial, ER-mediated process of protein glycosylation. Our data showed that PA:OA treatment significantly increases IRE1α (inositol-requiring enzyme 1 α) (Fig. 2A), CHOP (C/EBP homologous protein) (Fig. 2B), BIP (binding immunoglobulin protein) (Fig. 2C) and BAX (Bcl-2 Associated X-protein) (Fig. 2D) mRNA expression levels compared to control cells (p < 0.001). Notably, ALA treatment (both 1 and 5 µM) was able to revert this effect, resulting in a significant decrease in IRE1α, CHOP, BIP and BAX mRNA expression levels, compared to PA:OA-treated cells, when co-treated with PA:OA, however when added alone it was able to determine an increase in IRE1alpha, BIP and BAX (Additional file 2: Fig. S2A, C, D) and a reduction in CHOP (Additional file 2: Fig. S2B), compared to untreated cells.

**Fig. 3:**
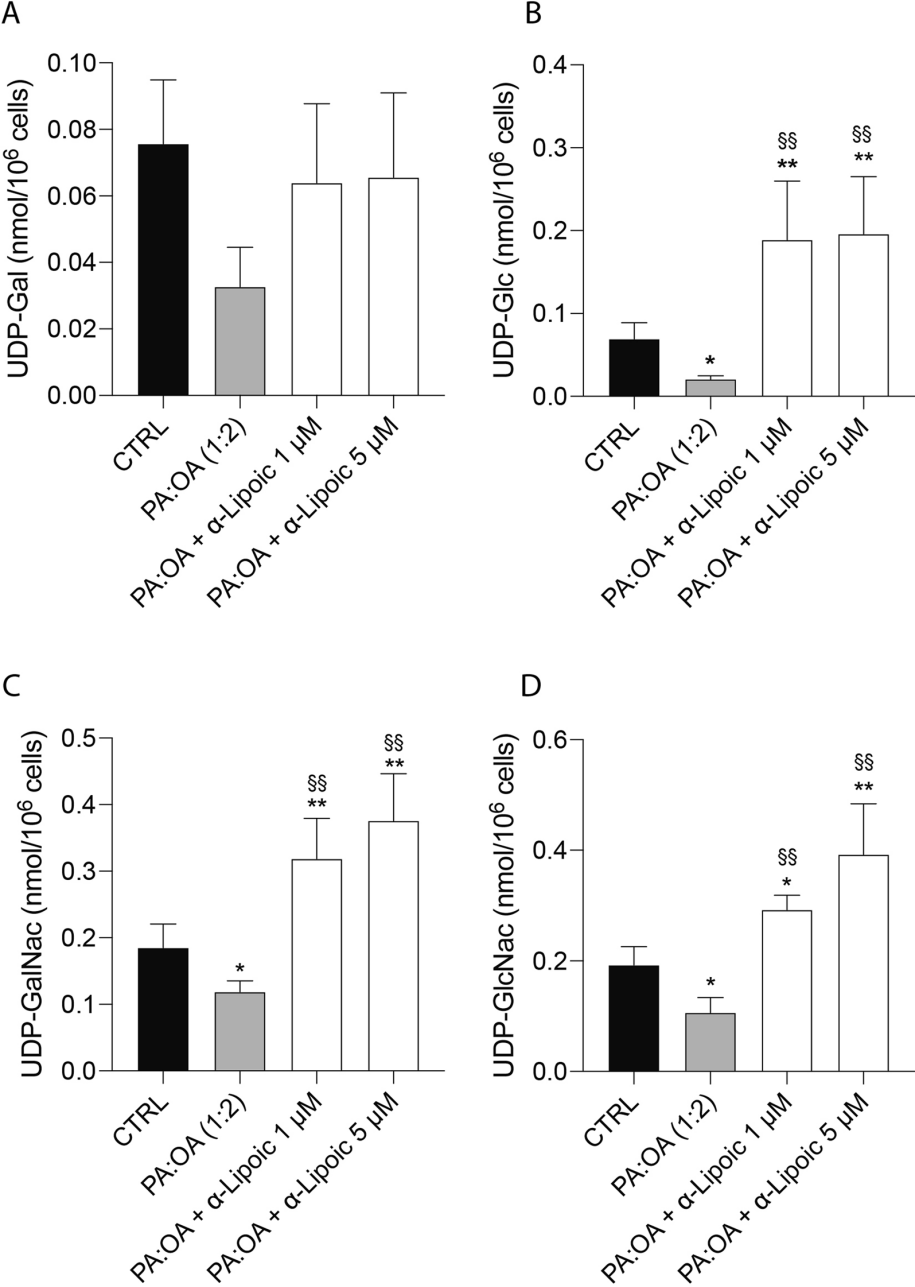
ALA restores the effect of palmitic acid/oleic acid on metabolites of the hexosamine biosynthetic pathway. (**A**) UDP-Galactose (UDP-Gal). **B** UDP-Glucose (UDP-Glc) **C** UDP-N-acetyl-galactosamine (UDP-GalNac). **D** UDP-N-acetyl-glucosamine (UDP-GlcNac). Values represent the mean ± SD of 6 independent experiments. * vs CTRL (* p < 0.05, ** p < 0.01); ^§^ vs PA:OA (^§§^ p < 0.01)

As shown in Fig. 3, treatment with PA:OA caused a dramatic decrease in the concentration of the UDP-derivatives involved in the ER-mediated process of protein glycosylation (UDP-Gal. UDP-Glc, UDP-GalNac, UDP-GlcNac), particularly evident when considering UDP-GalNac, UDP-GlcNac, i.e., the two metabolites directly involved in the donation of the carbohydrate moiety for protein glycosylation. The addition of ALA following the challenge with PA:OA, significantly increased the concentration of these compounds, to levels almost double those measured in untreated cells, strongly suggesting the return to a correct ER functioning in the protein glycosylation process.

### ALA enhances the antioxidant response and HO-1 expression in palmitic and oleic acid treated HepG2 cells

Since ER stress has been linked to oxidative stress in the pathophysiology of numerous diseases and since the protein folding process depends on redox homeostasis, we also evaluated oxidative stress.

**Fig. 4:**
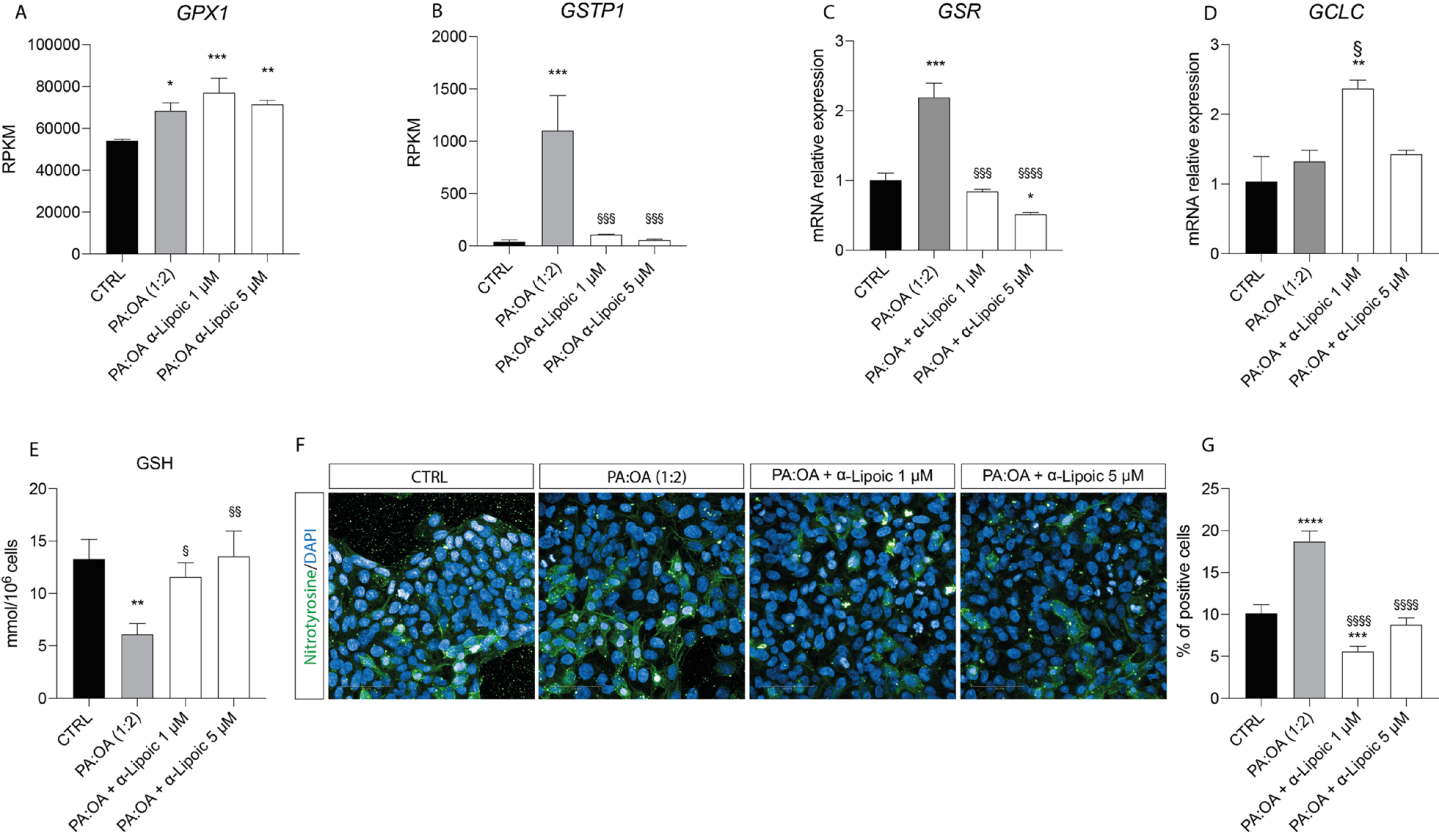
Effect of ALA on oxidative stress markers. Expression levels of **A** GPX1, **B** GSTP1, **C** GSR and **D** GCLC. **E** GSH levels. **F** Nitrotyrosine staining. **G** % of nitrotyrosine positive cells. Values represent the mean ± SD of experiments performed in quadruplicate. * vs CTRL (*p < 0.05, **p < 0.01, ***p < 0.001, ****p < 0.0001); § vs PA:OA (§p < 0.05, §§p < 0.01, §§§p < 0.001, §§§§p < 0.0001). Scale bars in (F) 50 μm

Our results showed that PA:OA causes a significant increase in oxidative stress of HepG2 cells, as shown by the increase in the expression levels of GPX1 (Glutathione Peroxidase 1) (Fig. 4A), GSTP1 (Glutathione S-Transferase Pi 1) (Fig. 4B), GSR (glutathione-disulfide reductase) (Fig. 4C), compared to control cells (p < 0.05). Interestingly, ALA was able to reverse the effect of PA:OA on gene expression of GSTP1 and GSR, resulting in a significant decrease (p < 0.001) of both genes (at the two concentrations used), compared to PA:OA-treated cells (Fig. 4B, C). Furthermore, ALA treatment (at concentration of 1 µM) was able to induce a significant increase in the expression of GCLC (Glutamate-Cysteine Ligase Catalytic Subunit) compared bot control and PA:OA-treated cells (Fig. 4D).

In this regard we measured GSH levels by HPLC analysis, and results confirmed that PA:OA induced a decrease in GSH levels in HepG2 cells and that ALA was able to significantly increase GSH levels (p < 0.05) compared to PA:OA treated cells (Fig. 4E). Finally, these data were confirmed by immunocytochemistry analysis, showing that in PA:OA-treated cells there is an increase in the expression of Nitrotyrosine, compared both control and ALA-treated cells (Fig. 4F), suggesting that ALA confers protection from oxidative stress to HepG2 cells treated with palmitic acid and oleic acid.

**Fig. 5:**
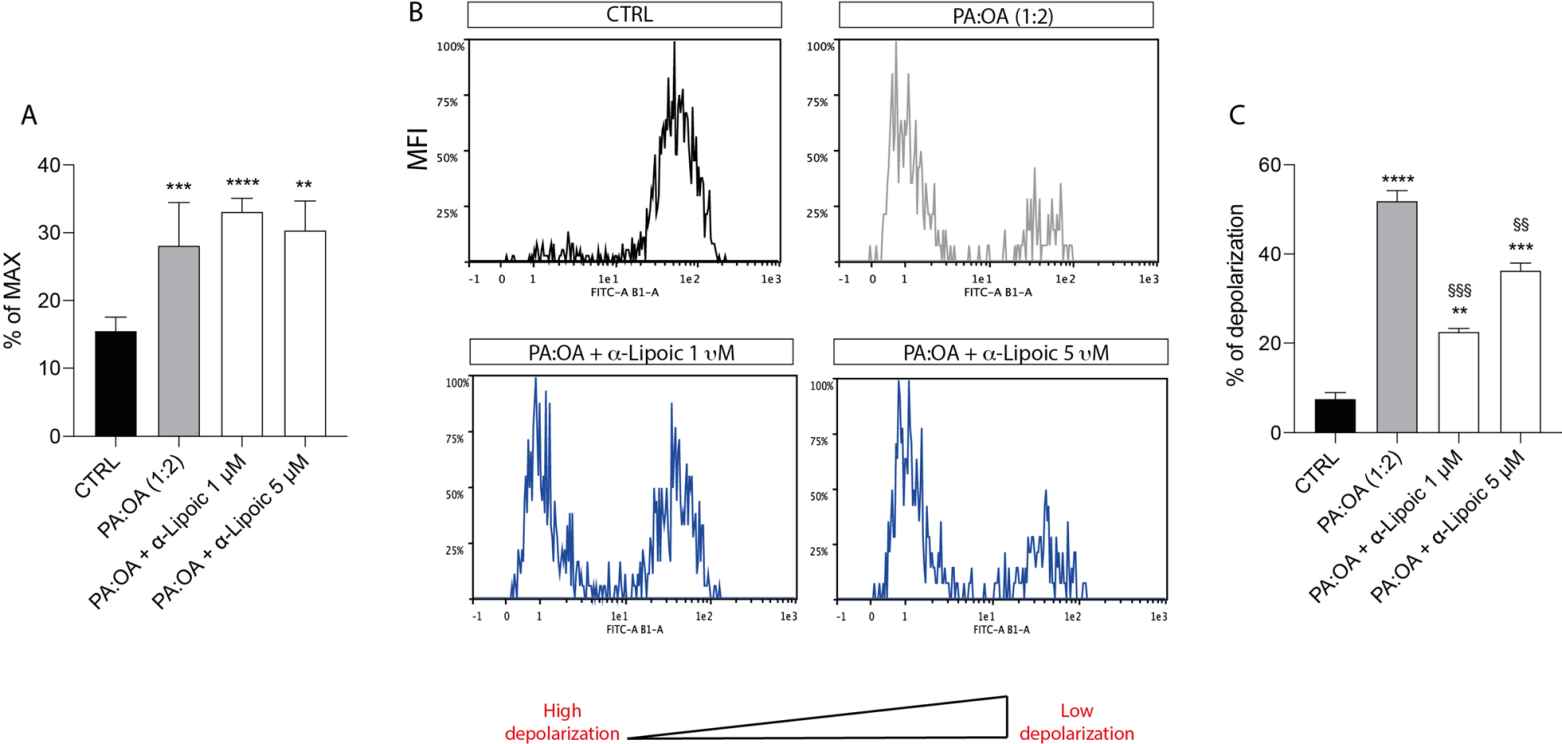
ALA decreases mitochondrial membrane potential following PA:OA treatment. Cytofluorimetric analysis of mitoSox and DiOC2 following 6 h of ALA treatment. **A** Quantification of % of mitoROS formation following 6 h of treatment. **B** Representative plots of mitochondrial depolarization following 6 h of treatment. **C** Quantification of % depolarization. Values represent the mean ± SD of experiments performed in quadruplicate. * vs CTRL (**p < 0.01, ***p < 0.001, ****p < 0.0001); ^§^ vs PA:OA (^§§^p < 0.01, ^§§§^p < 0.001)

We carried out a cytofluorimetric measurement of mitoROS (Fig. 5). Interestingly, following 6 hsr of treatment our results showed that PA:OA resulted in a significant increase of mitoROS formation. This increase was further increased by ALA treatment (both 1 and 5 µM) (Fig. 5A). Noteworthy, the increase in mitoROS was accompanied by a decrease of mitochondrial membrane potential (Fig. 5B, C) in the presence of PA:OA treatment alone, whereases ALA was able to decrease mitochondrial depolarization.

**Fig. 6:**
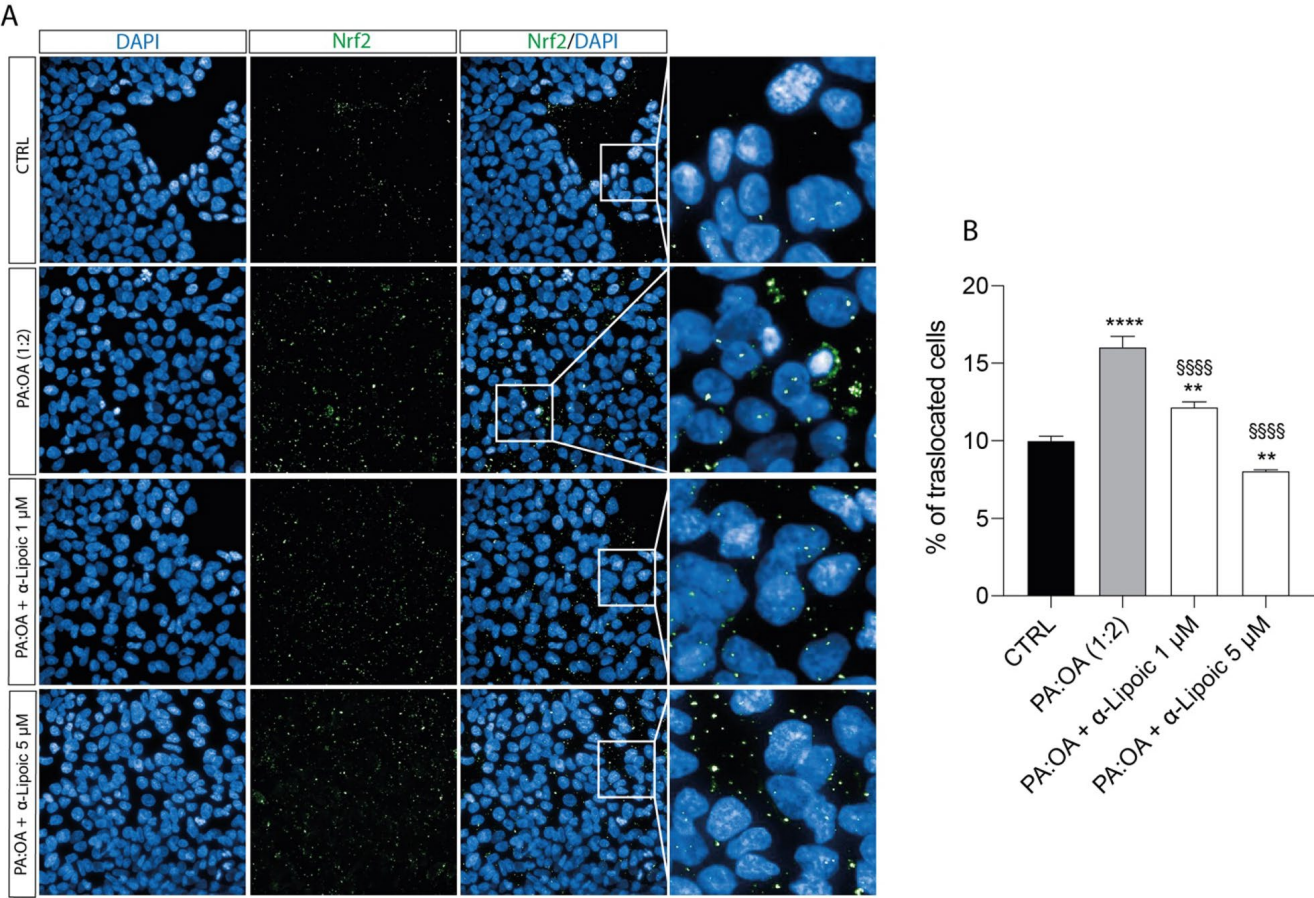
Effect of ALA on Nrf2 expression. **A** Immunocytochemistry for Nrf2. (**B**) % of nrf2 nu-clear translocated cells. Values represent the mean ± SD of experiments performed in quadruplicate. * vs CTRL (**p < 0.01, ****p < 0.0001); ^§^ vs PA:OA (^§§§§^p < 0.0001). Scale bars in **A** 50 μm

Consistently, the increased oxidative stress leads to an increase in Nrf2 nuclear translocation. In particular, PA:OA treatment results in a significant increase in the % of Nrf2 nuclear translocated cells compared to control cells (p < 0.0001) (Fig. 6A, B). Furthermore, both concentration of ALA resulted in a significant decrease in the % of Nrf2 nuclear translocated cells compared to PA:OA treated cells, but it has no effect when added alone (Additional file 3: Fig. S3A, B).

**Fig. 7:**
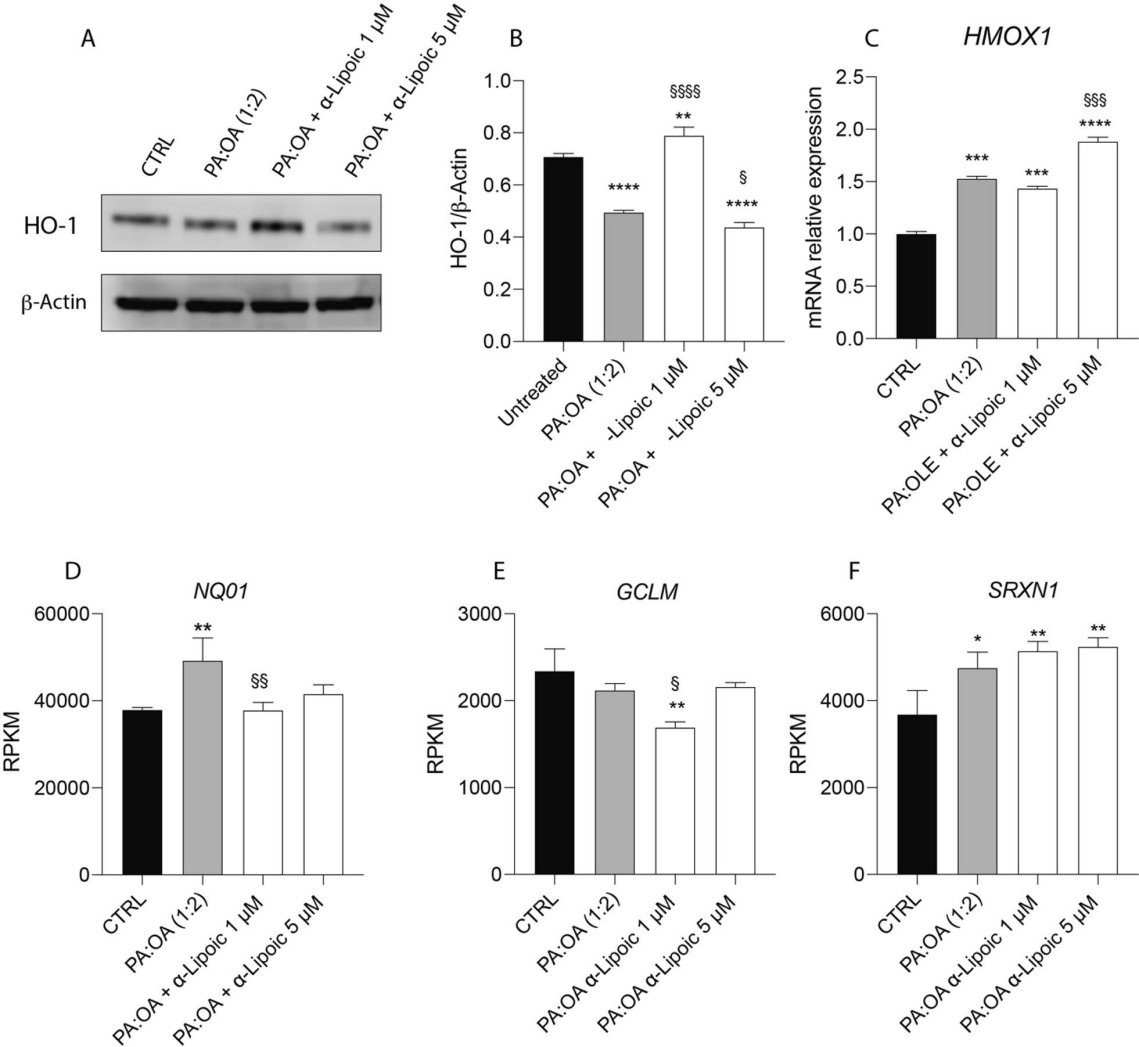
Effect of ALA on HO-1 and Nrf2 target genes expression. (**A**) Representative blots of HO-1. (**B**) Densitometric analysis of HO-1. (**C**) mRNA expression levels of HMOX1. Expression levels of (**D**) NQ01 (**E**) GCLM and (**F**) SRXN1. Values represent the mean ± SD of experiments performed in quadruplicate. * vs CTRL (*p < 0.05, **p < 0.01, ***p < 0.001, ****p < 0.0001); ^§^ vs PA:OA (^§^p < 0.05, ^§§^p < 0.01, ^§§§^p < 0.001, ^§§§§^p < 0.0001)

We further assess gene and protein expression of a well-established target of Nrf2 transcription activity. Interestingly, this set of experiments showed that HMOX1 expression was up-regulated following PA:OA treatment and this effect was not reverted by ALA treatment (Fig. 7C). On the other hand, protein expression showed a significant decrease following PA:OA treatment (Fig. 7A, B) compared to control cells (p < 0.0001) and a significant increase following ALA treatment, also at the concentration of 1 µM (Fig. 7A, B). Interestingly, ALA treatment alone in HepG2 cells was able to induce a significant increase in HO-1 protein expression compared to untreated cells (Additional file 3: Fig. S3C). Furthermore, gene expression analysis showed that treatment with PA:OA resulted in an increase in NQ01 expression compared to control cells, and that lipoic acid at a concentration of 1 µM was able to terminate a reduction of expression compared to PA:OA-treated cells (Fig. 7D). At the same time, PA:OA resulted in an increase in SRXN1 expression levels compared to control cells, and ALA treatment further increased its expression compared to untreated cells (Fig. 7F). While, PA:OA did not determine significant effects on the expression of GCLM, but lipoic acid (1 µM) determined a significant reduction in its expression compared to both control cells and PA:OA-treated cells (Fig. 7E).

### ALA improves the effect of palmitic acid/oleic acid on inflammation.

**Fig. 8:**
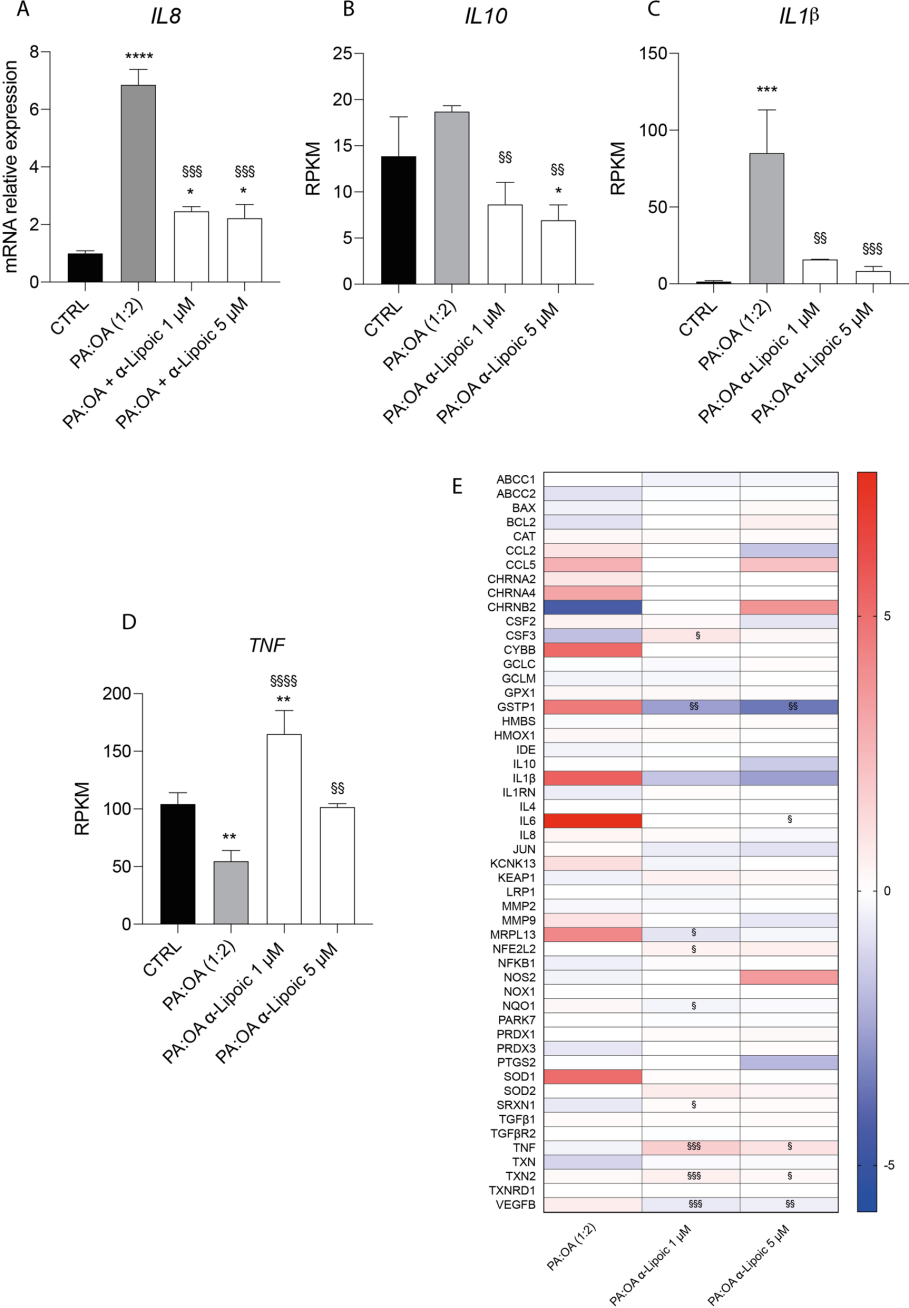
Effect of ALA in inflammatory cytokines expression. Expression levels of **A** IL8, **B** IL10, **C** IL1β and **D** TNF. **E** Heatmap of expression genes analyzed. Values represent the mean ± SD of experiments performed in quadruplicate. * vs CTRL (*p < 0.05, ** p < 0.01, ***p < 0.001, ****p < 0.0001); § vs PA:OA (§p < 0.05, §§p < 0.01, §§§p < 0.001, §§§§p < 0.0001)

Given the effect of PA:OA and ALA treatments on oxidative stress, we studied the effect on inflammation. In particular, we performed gene expression analysis of different cytokines, showing that PA:OA induced a significant increase in the expression of IL8 (Fig. 8A) and IL1beta (Fig. 8C) and that this effect was significantly reverted by ALA treatment (p < 0.001). Moreover, ALA was able also to induce a significant decrease in the expression of IL10 (Fig. 8B) compared to PA:OA-treated cells.

Consistently, PA:OA in Hepg2 cells results in a significant decrease of TNF (Fig. 8D), also in this case reverted by ALA treatment (at both concentration) (p < 0.01). Furthermore, DEG shows the expression of CHRNB2 seems to be inversely proportional to the concentration of ALA: the higher the concentration, the lower the expression of the gene encoding the cholinergic receptor subunit.

From the DEG (Fig. 8E) it is possible to notice the following significant variations in the expression of some genes compared to cells treated with the PA:OA mixture alone: there is a reduced expression of the GSTP1 gene, moreover IL6 falls within physiological ex-pression levels when cells are treated with ALA 5 μM. MRPL13 is also under expressed in cells treated with ALA 1 μM while NFE2L2 is expressed more in cells treated with ALA 1 μM. The 1 μM mixture also causes an increase in SRXN1 expression while both ALA 1 μM and ALA 5 μM cause significant overexpression in TNF and TXN2 as well as an under expression of VEGFB.

### ALA supplementation restores the effect of palmitic and oleic acid on senescence in HepG2 cells

**Fig. 9:**
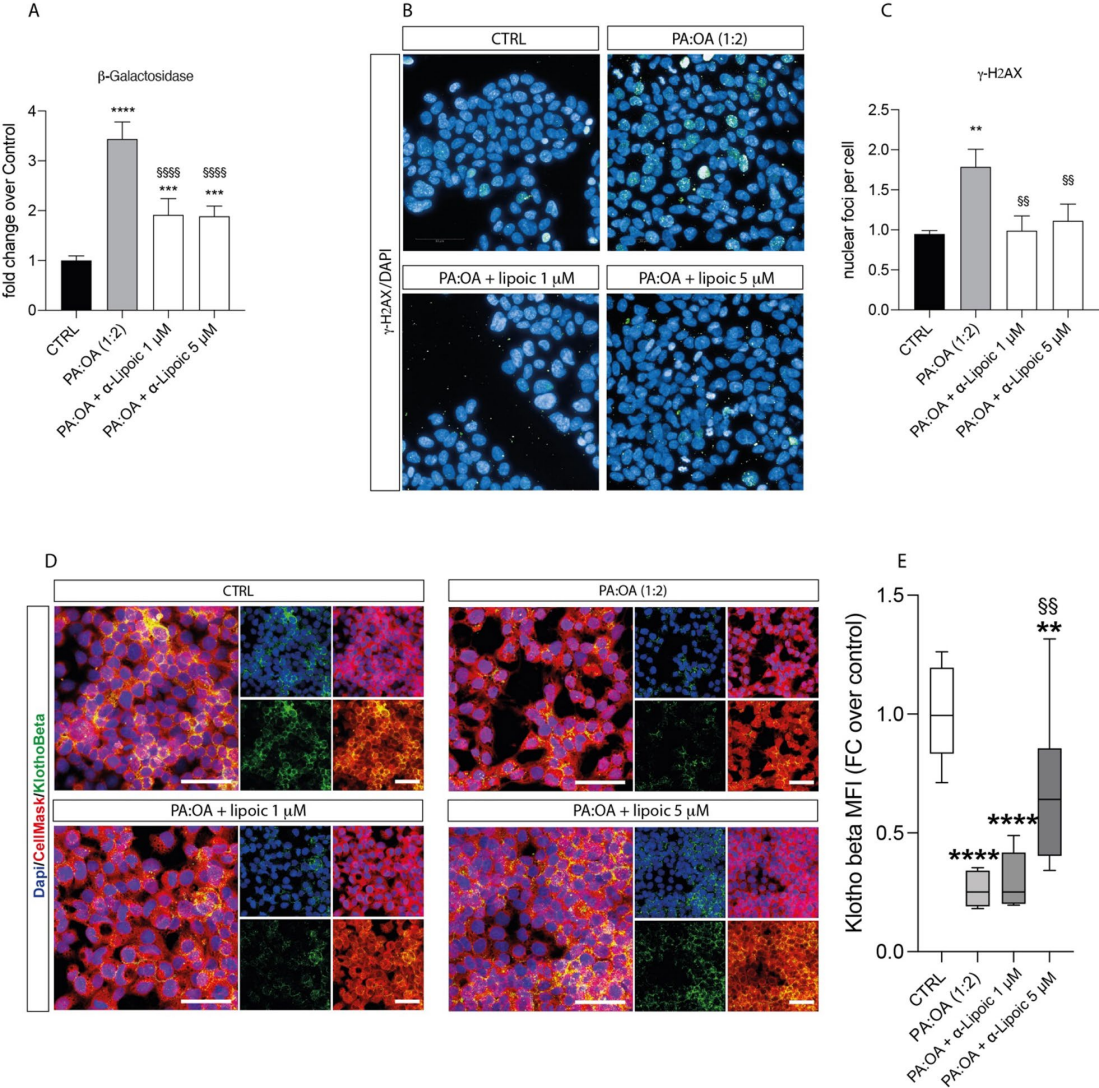
ALA reverses the effect of palmitic and oleic acid on senescence in HepG2 cells. **A** β-galactosidase, **B**, **C** γ-H2AX and **D** Klotho-Beta. Values represent the mean ± SD of experiments performed in quadruplicate. * vs CTRL (**p < 0.01, ***p < 0.001, ****p < 0.0001); § vs PA:OA (§§p < 0.01, §§§§p < 0.0001). Scale bars in **B** 50 μm

Consistently, β-galactosidase and γ-H2AX, two well established senescence markers, are significantly increased following PA:OA treatment (p < 0.01) (Fig. 9A–C), and ALA treatment alone has no significant effect in HepG2 (Additional file 4: Fig. S4), but restores the effect in PA:OA-treated cells, showing a significant decrease both in beta-galactosidase and gammaH2Ax, compared to PA:OA-treated cells (Fig. 9A–C). Our data were confirmed by Klotho-beta staining, another marker of cellular senescence. In particular, Fig. 9D shows that PA:OA treatment was able to induce a significant decrease in Klotho-beta expression, compared to control cells, and that treatment with lipoic acid (5 µM) was able to reverse this effect, showing an increase in expression when compared to HepG2 cells treated with PA:OA (Fig. 9D, E) (Additional file 5).

## Discussion

**Fig. 10:**
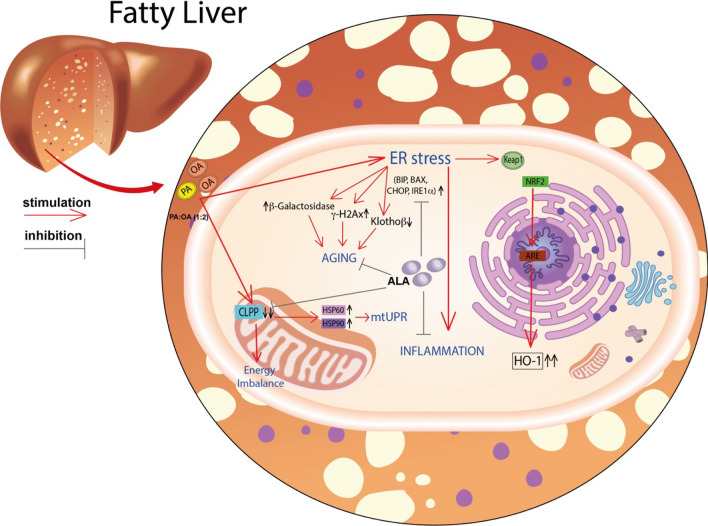
Representative scheme of the pathophysiological model described and the effects of lipoic acid

NAFLD is a complex metabolic condition characterized by oxidative stress, fibrosis, and insulin resistance [25, 26]. The complex pathophysiology of this condition involves mitochondrial homeostasis, which appears to play a major role. In the context of steatosis, mitochondrial dysfunction can occur due to various factors such as oxidative stress, lipid overload, and impaired mitochondrial metabolism [27] (Fig. 10). This dysfunction can trigger the UPRmt as a protective mechanism to restore mitochondrial protein homeostasis. The UPRmt promotes the expression of specific genes involved in mitochondrial protein folding, degradation, and quality control. Previous studies showed a potential link between the UPRmt and steatosis. In animal models and human samples of steatosis, there is evidence of UPRmt activation [28]. The accumulation of unfolded or misfolded proteins within the mitochondria in steatosis can activate the UPRmt pathway aiming to restore mitochondrial protein homeostasis and alleviate cellular stress caused by impaired mitochondrial function [5].

HSPs, particularly HSP60 and HSP90, have been implicated in the pathogenesis of NAFLD [29, 30]. HSP60 is a chaperone protein located in the mitochondria, where it assists in protein folding and prevents protein aggregation. In NAFLD, mitochondrial dysfunction occurs, leading to an accumulation of unfolded or misfolded proteins. This can trigger the activation of the unfolded protein response (UPR) and the upregulation of chaperone proteins like HSP60 [28]. Increased expression of HSP60 has been observed in animal models and human samples of NAFLD, suggesting its involvement in the cellular response to mitochondrial stress and dysfunction [31]. HSP90 is another important chaperone protein that assists in the folding and stabilization of client proteins involved in various cellular processes, including signal transduction and protein degradation. HSP90 has been implicated in the regulation of lipid metabolism and inflammation, both of which are dysregulated in NAFLD [32, 33]. Studies have shown that HSP90 interacts with key signaling molecules involved in lipid metabolism, such as AMP-activated protein kinase (AMPK) and peroxisome proliferator-activated receptors (PPARs), which are critical regulators of lipid homeostasis [34]. Disruption of the HSP90-mediated protein folding machinery can contribute to metabolic disturbances seen in NAFLD. Consistently, our results showed that cell treatment with PA:OA resulted in a significant increase of both HSP60 and HSP90 which was significantly reduced by lipoic acid treatment (Fig. 10). Similarly, studies using animal models have demonstrated alterations in CLPP expression and activity in the liver during NAFLD development [35]. In certain NAFLD models, CLPP expression was found to be downregulated, potentially impairing mitochondrial protein quality control mechanisms. Reduced CLPP levels may result in the accumulation of damaged or misfolded proteins within the mitochondria, contributing to mitochondrial dysfunction and oxidative stress in NAFLD [6, 35]. Furthermore, studies utilizing genetic knockout or overexpression models of CLPP have provided insights into its role in NAFLD pathogenesis. For instance, enhancing CLPP expression in a mouse model of NAFLD was shown to improve mitochondrial function and attenuate liver injury. Conversely, CLPP deficiency exacerbated mitochondrial dysfunction and liver inflammation in a high-fat diet-induced NAFLD model [36, 37]. Consistently, our results showed that cell treatment with PA:OA resulted in a significant decrease of CLPP which was restored by lipoic acid treatment. Our results are also consistent with increased ER stress following PA:OA treatment. Our experimental conditions disrupt ER homeostasis and lead to the accumulation of unfolded or misfolded proteins within the ER lumen (Fig. 10). Consequently, the unfolded protein response (UPR), a cellular stress response pathway, is activated to restore ER function and protein homeo-stasis. However, prolonged or severe ER stress can lead to UPR dysfunction and contribute to liver injury and inflammation observed in NAFLD [12, 38]. ER stress in NAFLD promotes lipogenesis, impairs lipid metabolism, and disrupts insulin signaling pathways, contributing to the development of hepatic steatosis (accumulation of fat in the liver) and the progression of NAFLD to more severe forms, such as non-alcoholic steatohepatitis (NASH) and fibrosis [39–41].

During ER stress, specific signaling pathways are activated to restore ER homeostasis. The HBP is one of these metabolic pathways because of its strict connection in providing substrates (UDP-derivatives) for protein glycosylation. Our data clearly demonstrate that NAFLD provokes an imbalance in this fundamental cell process through the dramatic depletion of the UDP-derivatives concentrations, a phenomenon well known to be associated with ER-stressing conditions [42]. Treatment with ALA positively improved levels of UDP-derivatives, presumably through the general amelioration of energy metabolism (unpublished results) that renders possible the restoration of correct concentrations of the substrates needed for O- and N-glycosylation of proteins. In PA:OA-treated cells, we also found the activation of chaperone proteins, such as BiP/GRP78, which assist in protein folding, and the upregulation of genes involved in ER-associated degradation (ERAD) to clear misfolded proteins [43] (Fig. 10). However, in NAFLD, the sustained activation of the UPR and ER stress can overwhelm the cellular defense mechanisms, leading to cellular dysfunction and liver damage [44]. Our results showed a significant increase of ER stress which was reduced by lipoic acid treatment. Moreover, ER stress in NAFLD can trigger inflammatory responses and oxidative stress. It promotes the production of pro-inflammatory cytokines and chemokines, leading to the recruitment of immune cells and further exacerbating liver inflammation [45]. Our results are consistent with in vivo results showed that ALA treatment resulted in a significant decrease of ER and oxidative stress in an animal model of NAFLD [46]. To this regard, our data showed that PA:OA treatment results in a significant increase of mitoROS formation also following ALA treatment (1µM and 5µM). To this regard previously reports, showed that mitochondrial ROS are chemical species involved in mitohormesis. In particular, an increase in mitochondrial ROS, secondary to a stressful event, leads to an increase mitochondrial biogenesis. Consistently, we have previously showed that ALA 1 and 5 µM resulted in a significant restore of mitochondrial membrane potential and biogenesis [47]. Therefore, it is conceivable that increased mitoROS may be the results of increased mitochondrial metabolism.

Consistently, increased oxidative stress in turn leads to the activation of Nrf2 nuclear translocation with concomitant transcription of its target genes (i.e. heme oxygenase-1) [48] (Fig. 10).

This inflammatory response contributes to the progression of NAFLD and the development of more severe liver conditions [49]. Consistently, DEG showed a class of gene differentiating the metabolic status after treatment with PA:OA. In particular, PA:OA results in a significant activation of a pathway involving CYBB, GSTP1, SOD1, IL6 and IL1b [50–52] which was reversed by ALA treatment (both 1 and 5 µM). Consistently, Thioredoxin-2 overexpression reduces mitochondrial oxidative stress and apoptosis while inhibiting VEGF-B signaling prevents the development of NAFLD by targeting lipolysis in white adipose tissue [53]. Inflammatory processes and metabolic dysregulation, along with additional molecules and pathways, such as the UPRmt [54, 55], as well as mitochondrial proteases and chaperones like heat shock protein 60 [56], ClpP [57], among others, have demonstrated an association between mitochondrial dysfunction, senescence, and chronic liver disease. Previous clinical studies strongly support the involvement of cellular senescence in NAFLD. To this regard, previous studies indicate the overexpression of anti-apoptotic BCL family proteins [58], the presence of the DNA damage marker (γ-H2AX) [59, 60], and metabolic changes, including the activation of β-galactosidase (senescence-associated β-galactosidase, SA-β-gal) [61]. Moreover, there is evidence suggesting that obesity can induce resistance to FGF21 [62], and obese mice exhibit reduced Klotho beta expression [63–65]. This implies that diminished Klotho beta expression may be an underlying cause of FGF21 resistance and the development of NAFLD. Consistently, our results demonstrate a significant increase in β-galactosidase, γ-H2AX, and Klotho beta expression in HepG2 cells treated with PA:OA, which was reversed by ALA treatment thus suggesting that such pharmacological treatment has a significant impact as a senolytic-like agent [66–68].

Similarly, ER stress resulted in a significant increase of Nrf2 activation and increased expression of one its targeted gene (i.e. heme oxygenase-1) [17]. Lipoic acid resulted in a significant decrease of the inflammatory response and of the activation of Nrf2 with a concomitant reduction of heme oxygenase-1 expression (Fig. 10).

## Study limitations and strengths

The primary limitation of the current study is its reliance on evidence demonstrating that ALA's positive impact is observed solely in an in vitro model. Notably, NAFLD is a multifaceted condition encompassing critical metabolic components like insulin resistance, systemic inflammation, and oxidative stress. Although previous studies have hinted at the potential benefits of ALA treatment in clinical trials, they have fallen short in establishing a direct influence of ALA on hepatocyte metabolism and lipotoxicity.

Consequently, the key strength of this manuscript lies in its ability to assess the direct impact of ALA in an in vitro NAFLD model, thus unravelling potential and additional mechanisms that contribute to ALA's favorable effect on this condition.

## Conclusions

In conclusion, there is a significant association between steatosis and mitochondrial unfolded protein response. Alterations in such mechanism results in a significant ER stress response and concomitant inflammation and oxidative stress. Our data, further suggest the beneficial effects of ALA treatment in NAFLD with particular regards to direct effect of such compound on hepatocytes in addition to its indirect effects (i.e. improvement of insulin resistance, systemic inflammation and oxidative stress). While more research is needed to specifically investigate the effects of lipoic acid on mitochondrial unfolded protein response, its impact on ER stress, inflammation and oxidative stress suggests that it may play a role in supporting optimal mitochondrial proteostasis. Understanding the underlying mechanisms involved in these processes may provide insights into the development of therapeutic strategies for the treatment of steatosis.

### Supplementary Information

**Additional file 1. **Effect of ALA on unfolded protein response in HepG2 cells. (A) Western blot analysis of HSP90, HSP60 and CLPP proteins. (B) Densitometry analysis of HSP90. (C) Densitometry analysis of HSP60. (D) Densitometry analysis of CLPP. β‐Actin protein was used as total protein loading reference. Values represent the mean ± SD of experiments performed in quadruplicate. * vs Untreated (* p < 0.05, ** p < 0.01)

**Additional file 2. **Effect of ALA on ER stress in HepG2 cells. mRNA expression levels of (A) IRE1α, (B) CHOP, (C) BIP and (D) BAX. b-Actin gene was used as housekeeping gene. Values represent the mean ± SD of experiments performed in quadruplicate. * vs Untreated (* p < 0.05, ** p < 0.01, *** p < 0.001).

**Additional file 3. **Effect of ALA on Nrf2 and HO-1 expression. (A) Immunocytochemistry for Nrf2. (B) % of nrf2 nuclear translocated cells. (C) Western blot analysis of HO-1 protein expression. Values represent the mean ± SD of experiments performed in quadruplicate. * vs Untreared (* p < 0.05, *** p < 0.001).

**Additional file 4. **Effect of ALA on Nitrotyrosine and γ-H2AX in HepG2 cells. (A) Nitrotyrosine staining. (B-C) γ-H2AX staining and nuclear foci per cell quantification. (D) β-galactosidase.  Values represent the mean ± SD of experiments performed in quadruplicate. * vs CTRL (** p< 0.01). Scale bar in (A) and (B) 50 μm.

**Additional file 5. **Preliminary time-resolved citotoxicity dose-response curve to assess ALA concentration.

**Additional file 6: Table S1.** RPKM values obtained from sequencing.

## Data Availability

The datasets used and/or analysed in this study are reported within the manuscript and/or additional files and are available from the corresponding authors.

## Abbreviations

ALA: ( +)-Lipoic acid
AMPK: AMP-activated protein kinase
BAX: Bcl-2-associated X protein
BIP: Binding Immunoglobulin Protein
CHOP: C/EBP Homologous Protein
CLPP: Caseinolytic protease P
ER: Endoplasmic reticulum
ERAD: ER-associated degradation
GPX1: Glutathione peroxidase 1
GSH: Glutathione
GSR: Glutathione-disulfide reductase
GSTP1: Glutathione S-transferase pi 1
HSP60: Heat shock protein 60
HSP90: Heat shock protein 90
IRE1α: Inositol requiring enzyme-1
NAFLD: Non-alcoholic fatty liver disease
PA:OA: Palmitic acid:oleic acid
PPARs: Peroxisome proliferator-activated receptors
UDP-Gal: UDP-Galactose
UDP-GalNac: UDP-N-acetyl-galactosamine
UDP-Glc: UDP-Glucose
UDP-GlcNac: UDP-N-acetyl-glucosamine
UPRmt: Unfolded protein response in mitochondria
