# Supplementary figures and images for: (+)-Lipoic acid reduces mitochondrial unfolded protein response and attenuates oxidative stress and aging in an in vitro model of non-alcoholic fatty liver disease
Source: J Transl Med. 2024 Jan 20;22:82. doi: 10.1186/s12967-024-04880-x (PMC10799515; doi:10.1186/s12967-024-04880-x)

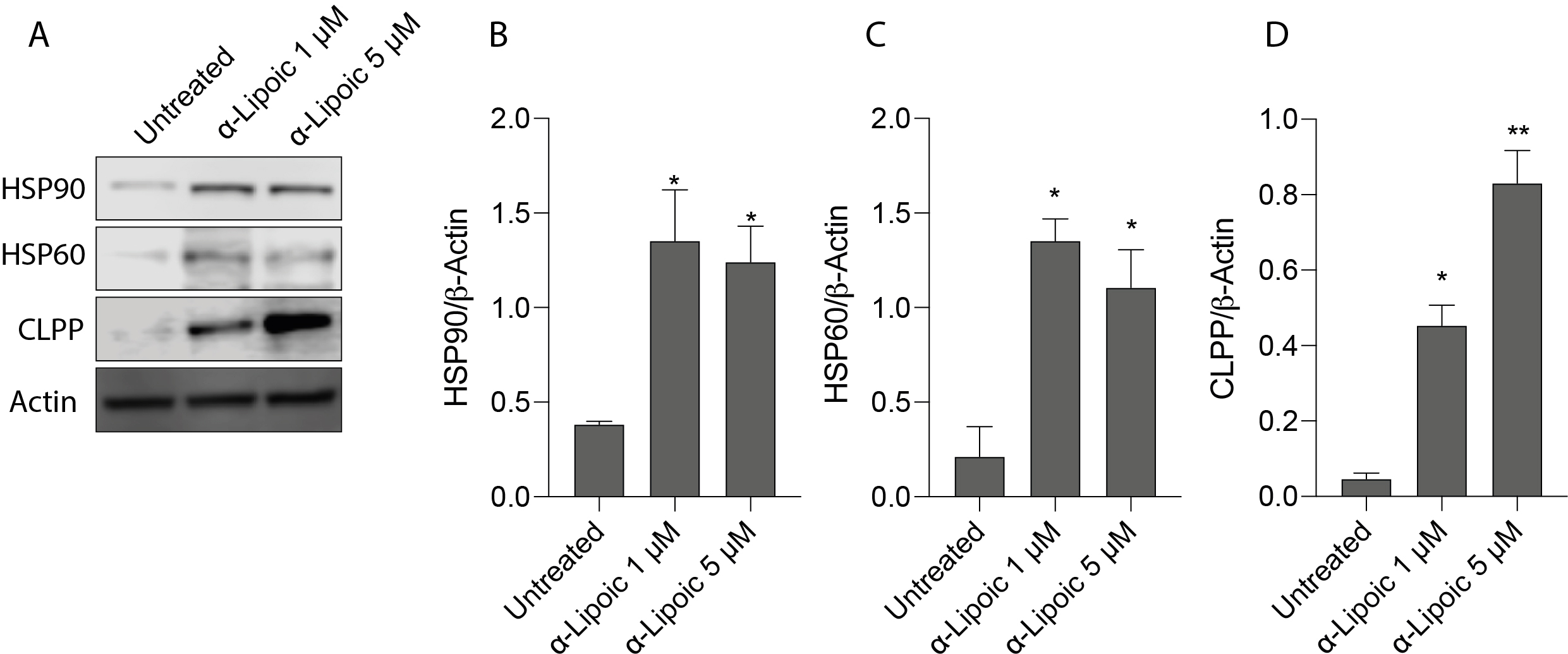

Supplement: Supplementary file 1 — Additional file 1. Effect of ALA on unfolded protein response in HepG2 cells. (A) Western blot analysis of HSP90, HSP60 and CLPP proteins. (B) Densitometry analysis of HSP90. (C) Densitometry analysis of HSP60. (D) Densitometry analysis of CLPP. β‐Actin protein was used as total protein loading reference. Values represent the mean ± SD of experiments performed in quadruplicate. * vs Untreated (* p < 0.05, ** p < 0.01) [file 12967_2024_4880_MOESM1_ESM.jpg]

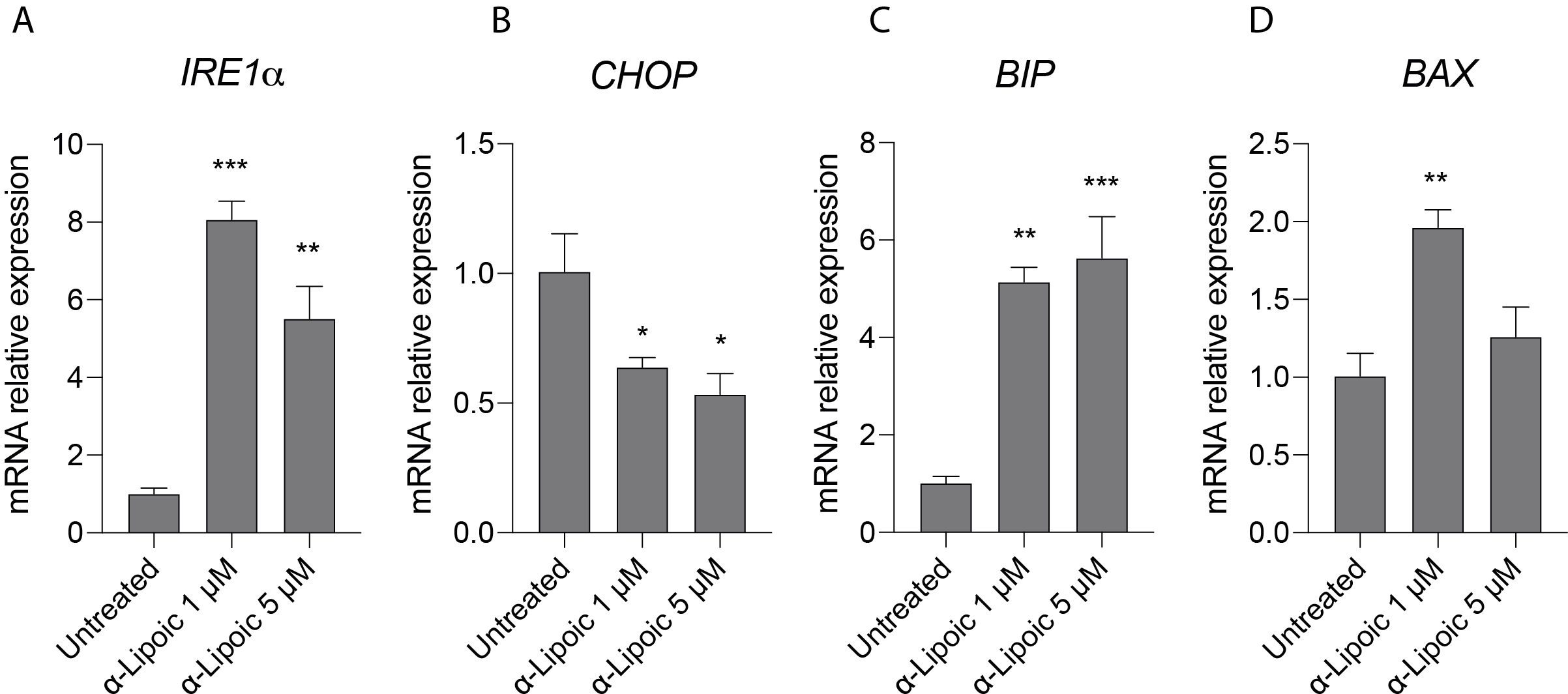

Supplement: Supplementary file 2 — Additional file 2. Effect of ALA on ER stress in HepG2 cells. mRNA expression levels of (A) IRE1α, (B) CHOP, (C) BIP and (D) BAX. b-Actin gene was used as housekeeping gene. Values represent the mean ± SD of experiments performed in quadruplicate. * vs Untreated (* p < 0.05, ** p < 0.01, *** p < 0.001). [file 12967_2024_4880_MOESM2_ESM.jpg]

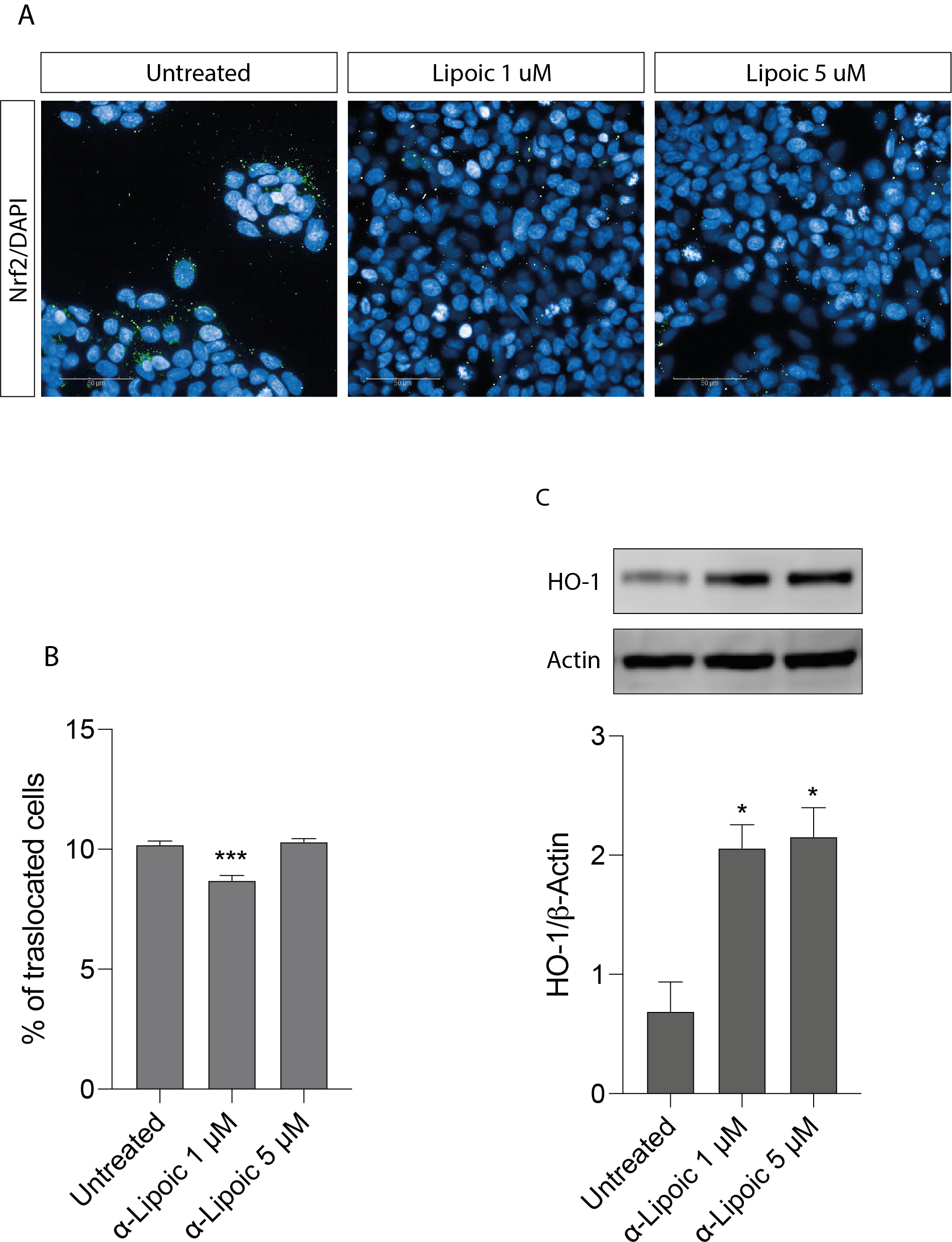

Supplement: Supplementary file 3 — Additional file 3. Effect of ALA on Nrf2 and HO-1 expression. (A) Immunocytochemistry for Nrf2. (B) % of nrf2 nuclear translocated cells. (C) Western blot analysis of HO-1 protein expression. Values represent the mean ± SD of experiments performed in quadruplicate. * vs Untreared (* p < 0.05, *** p < 0.001). [file 12967_2024_4880_MOESM3_ESM.jpg]

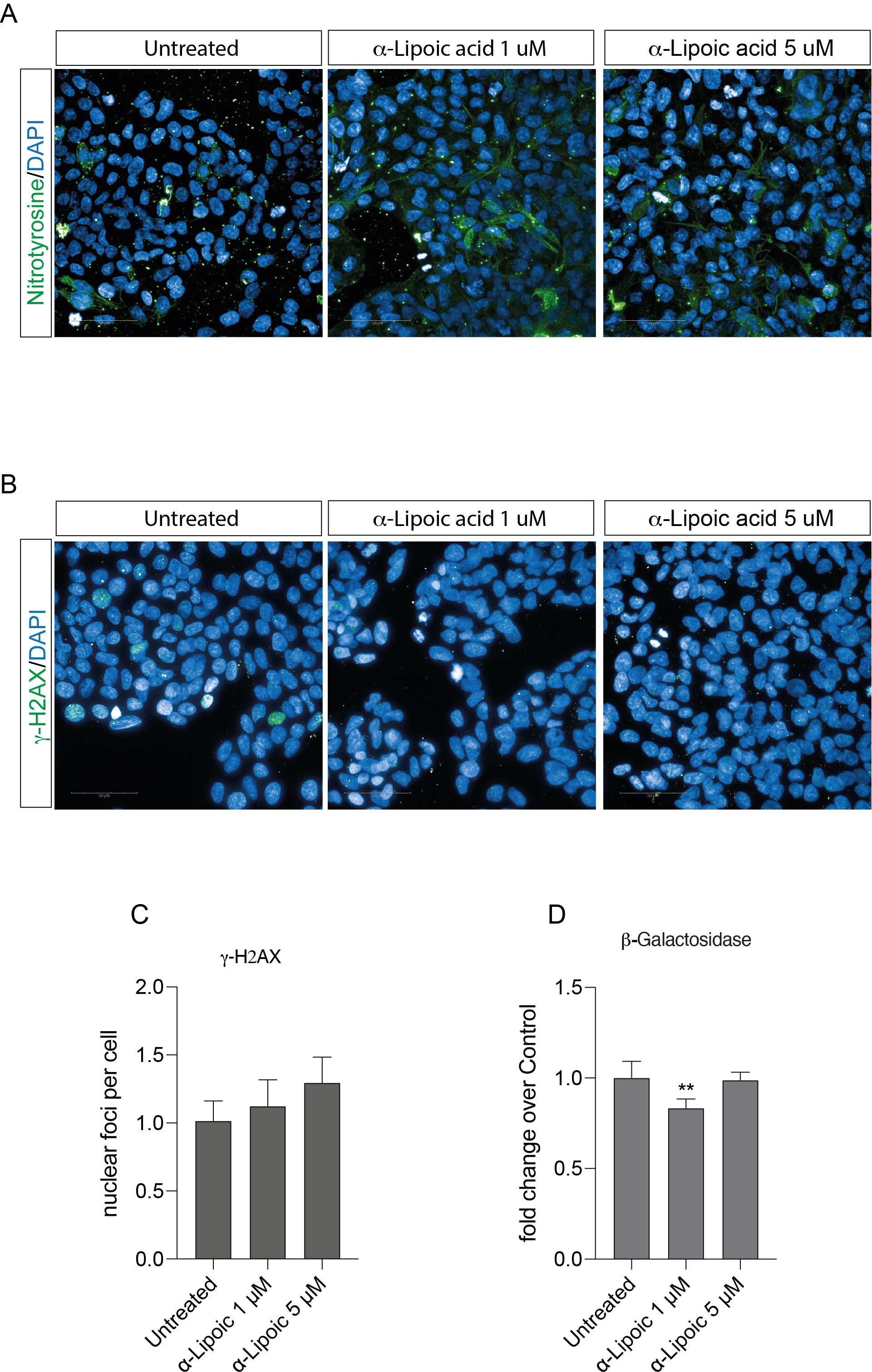

Supplement: Supplementary file 4 — Additional file 4. Effect of ALA on Nitrotyrosine and γ-H2AX in HepG2 cells. (A) Nitrotyrosine staining. (B-C) γ-H2AX staining and nuclear foci per cell quantification. (D) β-galactosidase. Values represent the mean ± SD of experiments performed in quadruplicate. * vs CTRL (** p< 0.01). Scale bar in (A) and (B) 50 μm. [file 12967_2024_4880_MOESM4_ESM.jpg]

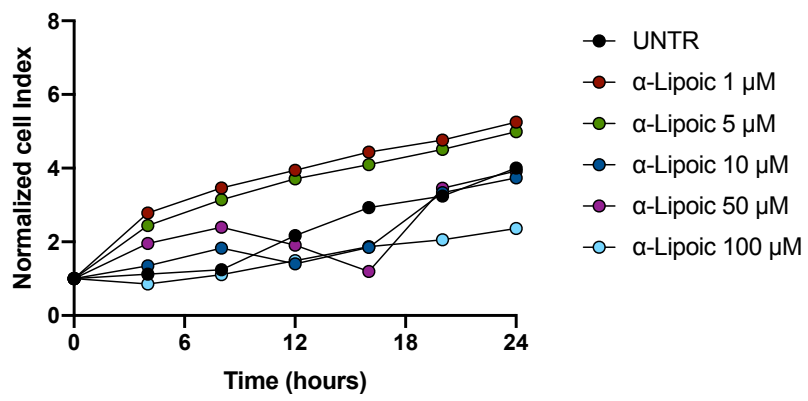

Supplement: Supplementary file 5 — Additional file 5. Preliminary time-resolved citotoxicity dose-response curve to assess ALA concentration. [file 12967_2024_4880_MOESM5_ESM.pdf]
