# Supplementary material for: (+)-Lipoic acid reduces mitochondrial unfolded protein response and attenuates oxidative stress and aging in an in vitro model of non-alcoholic fatty liver disease
Source: J Transl Med. 2024 Jan 20;22:82. doi: 10.1186/s12967-024-04880-x (PMC10799515; doi:10.1186/s12967-024-04880-x)
Supplement: Supplementary file 6 — Additional file 6: Table S1. RPKM values obtained from sequencing. [file 12967_2024_4880_MOESM6_ESM.pdf]

Table S1. RPKM values obtained from sequencing

Name	CTRL	CTRL	CTRL	PA:OA + α-Lipoic 1 µm	PA:OA + α-Lipoic 1 µm	PA:OA + α-Lipoic 1 µm	PA:OA + α-Lipoic 5µm	PA:OA + α-Lipoic 5µm	PA:OA + α-Lipoic 5µm	PA:OA	PA:OA	PA:OA
**GPX1**	54806,43	53479,69	54143,06	83989,36	70097,41	77043,39	73359,47	69733,66	71546,56	72228,92	64452,72	68340,82
**NQO1**	38468,81	37363,06	37915,94	39602,18	36045,72	37823,95	39432,21	43694,07	41563,14	44052,89	54418,03	49235,46
**SOD1**	38151,99	43025,63	40588,81	38172,44	45522,06	41847,25	40118,95	40012,89	40065,92	41485,91	38743,87	40114,89
**PARK7**	23868,72	27057,29	25463,01	21982,57	21761,71	21872,14	21701,4	22726,2	22213,8	25868,49	23327,1	24597,8
**TXN**	22864,96	22633,35	22749,16	25099,01	20375,54	22737,28	22376,48	25096,66	23736,57	21206,59	34342,55	27774,57
**PRDX3**	17277,44	17220,87	17249,15	16616,35	16304,13	16460,24	17394,77	18851,25	18123,01	15745,23	18152,92	16949,08
**HMOX1**	10690,71	11993,38	11342,05	15018,07	14398,36	14708,21	14065,69	13082,74	13574,21	14137,22	12472,08	13304,65
**TXN2**	9130,892	8987,884	9059,388	14334,42	11215,07	12774,75	10759	10203,18	10481,09	8778,076	9434,859	9106,467
**SOD2**	6946,713	6909,628	6928,17	6394,07	7775,051	7084,561	5831,835	5230,129	5530,982	5237,199	3753,088	4495,144
**GCLC**	5548,713	5586,15	5567,432	3259,549	5548,557	4404,053	5712,122	5587,535	5649,829	6253,247	4215,977	5234,612
**SRXN1**	4232,966	3136,911	3684,938	4917,154	5363,789	5140,472	5032,918	5449,179	5241,048	5115,102	4386,095	4750,599
**CAT**	3979,379	3545,419	3762,399	5930,653	5007,703	5469,178	5159,663	5920,622	5540,142	4489,161	5757,382	5123,271
**GCLM**	2596,908	2085,264	2341,086	1755,865	1628,91	1692,387	2104,548	2206,933	2155,74	2043,877	2195,373	2119,625
**IDE**	1588,177	1581,648	1584,912	1215,876	1068,35	1142,113	1249,253	1193,335	1221,294	1152,107	1485,134	1318,62
**PRDX1**	1520,371	1530,965	1525,668	1068,076	963,3559	1015,716	1122,688	1010,335	1066,511	900,1999	1033,547	966,8732
**HMBS**	1478,996	1508,422	1493,709	1490,005	1354,002	1422,003	1413,956	1551,318	1482,637	1355,302	1271,12	1313,211
**MRPL13**	183,9251	185,497	184,7111	87,2863	78,97632	83,13131	117,2711	125,1155	121,1933	134,5396	203,808	169,1738
**TNF**	114,164	94,70653	104,4353	144,9328	185,3745	165,1537	104,46	98,84666	101,6533	45,66953	63,994	54,83176
**GSTP1**	26,40229	59,36845	42,88537	113,2436	104,7659	109,0047	65,48257	48,54711	57,01484	1250	1337,192	1293,596

Oxidative Stress

Name	CTRL	CTRL	CTRL	PA:OA + α-Lipoic 1 µm	PA:OA + α-Lipoic 1 µm	PA:OA + α-Lipoic 1 µm	PA:OA + α-Lipoic 5µm	PA:OA + α-Lipoic 5µm	PA:OA + α-Lipoic 5µm	PA:OA	PA:OA	PA:OA
**BAX**	14222,6	13078,66	13650,63	12276,22	9639,263	10957,74	12533,39	12377,45	12455,42	11183,97	10530,54	10857,25
**LRP1**	1971,642	1952,663	1962,152	1338,392	1346,514	1342,453	1676,23	1803,19	1739,71	1567,646	1895,734	1731,69
**KEAP1**	1957,078	1652,697	1804,887	2227,384	1792,733	2010,058	1848,97	1818,419	1833,694	1506,014	1473,399	1489,706
**BCL2**	5,850628	0,828327	3,339477	4,06288	4,977772	4,520326	4,568165	4,987716	4,777941	3,994371	0	1,997185

Apopstpsis

Name	CTRL	CTRL	CTRL	PA:OA + α-Lipoic 1 µm	PA:OA + α-Lipoic 1 µm	PA:OA + α-Lipoic 1 µm	PA:OA + α-Lipoic 5µm	PA:OA + α-Lipoic 5µm	PA:OA + α-Lipoic 5µm	PA:OA	PA:OA	PA:OA
**TGFB1**	10787,01	10153,11	10470,06	11641,21	11473,66	11557,44	11908,12	12330,37	12119,25	10646,87	11714,68	11180,78
**IL8**	6485,508	7572,044	7028,776	7584,181	11018,77	9301,476	7521,909	6702,447	7112,178	9101,58	7766,556	8434,068
**TGFBR2**	5988,239	5494,747	5741,493	4352,197	4427,503	4389,85	4861,456	4504,333	4682,895	5202,066	4385,312	4793,689
**IL1RN**	3865,637	4030,603	3948,12	2894,214	2891,06	2892,637	2826,148	2851,919	2839,034	2980,818	2382,689	2681,753
**VEGFB**	3725,918	4740,185	4233,052	4172,499	3159,187	3665,843	3999,763	4082,263	4041,013	5172,438	6829,686	6001,062

**NFE2L2**	3427,974	3829,683	3628,828	3523,836	2994,311	3259,073	4084,785	3110,216	3597,501	2688,758	2318,055	2503,407
**NFKB1**	1910,771	1736,192	1823,481	1689,242	1585,457	1637,349	1601,612	1555,358	1578,485	1458,178	1697,002	1577,59
**CSF3**	309,7407	263,1169	286,4288	131,8624	203,786	167,8242	102,5214	122,4612	112,4913	89,64394	96,62517	93,13456
**CSF2**	84,00096	48,6523	66,32663	119,3179	139,225	129,2715	76,02232	48,82611	62,42422	107,5305	136,9782	122,2543
**IL10**	18,13754	9,629614	13,88358	6,29767	11,02257	8,66012	5,310665	8,590236	6,950451	19,34837	18,07448	18,71142
**CCL2**	7,986654	5,653718	6,820186	11,09242	14,56098	12,8267	6,235973	3,782611	5,009292	13,63172	31,83554	22,73363
**CCL5**	1,977295	0	0,988648	4,119309	7,209869	5,664589	46,31612	30,90383	38,60997	0	47,2901	23,64505
**IL1B**	0,965656	2,050751	1,508204	16,09404	15,84494	15,96949	11,30975	5,488206	8,398978	80	115,4758	97,73791

Immunoregulation and Infl

**Name**	**CTRL**	**CTRL**	**CTRL**	**PA:OA + α-Lipoic 1 µm**	**PA:OA + α-Lipoic 1 µm**	**PA:OA + α-Lipoic 1 µm**	**PA:OA + α-Lipoic 5µm**	**PA:OA + α-Lipoic 5µm**	**PA:OA + α-Lipoic 5µm**	**PA:OA**	**PA:OA**	**PA:OA**
**MMP2**	281,8157	205,9315	243,8736	598,4714	434,2408	516,3561	518,872	553,6958	536,2839	526,0041	753,7273	639,8657
**MMP9**	1,279825	0	0,639912	18,66386	9,333323	13,99859	11,99143	25,45814	18,72478	22,93643	61,218	42,07721

Metalloproteinase

**Name**	**CTRL**	**CTRL**	**CTRL**	**PA:OA + α-Lipoic 1 µm**	**PA:OA + α-Lipoic 1 µm**	**PA:OA + α-Lipoic 1 µm**	**PA:OA + α-Lipoic 5µm**	**PA:OA + α-Lipoic 5µm**	**PA:OA + α-Lipoic 5µm**	**PA:OA**	**PA:OA**	**PA:OA**
**CHRNB2**	2,54787	2,164352	2,356111	0	0,929037	0,464519	2,38725	0,724028	1,555639	0	0	0
**CHRNA2**	0,621812	0	0,310906	2,590847	0	1,295423	0,728265	0,883501	0,805883	0	7,435797	3,717899

naChR

Name	CTRL	CTRL	CTRL	PA:OA + α-Lipoic 1 µm	PA:OA + α-Lipoic 1 µm	PA:OA + α-Lipoic 1 µm	PA:OA + α-Lipoic 5µm	PA:OA + α-Lipoic 5µm	PA:OA + α-Lipoic 5µm	PA:OA	PA:OA	PA:OA
**ABCC2**	8862,512	7821,283	8341,898	7696,546	7534,891	7615,718	7978,945	7657,348	7818,146	8236,385	8920,618	8578,502
**ABCC1**	2882,319	3297,355	3089,837	2275,934	2012,607	2144,27	2348,2	2387,22	2367,71	3185,972	2638,282	2912,127
**KCNK13**	107,8747	108,2523	108,0635	167,9345	218,2858	193,1102	227,6946	220,6468	224,1707	188,1686	382,746	285,4573
**JUN**	20,70534	7,647246	14,17629	13,1282	16,41271	14,77046	8,434806	17,90732	13,17106	25,35271	10,76523	18,05897

Others
